# Potency ranking of pyrrolizidine alkaloids in metabolically competent human liver cancer cells and primary human hepatocytes using a genotoxicity test battery

**DOI:** 10.1007/s00204-023-03482-8

**Published:** 2023-03-16

**Authors:** Manuel Haas, Karina Wirachowski, Lea Thibol, Jan-Heiner Küpper, Dieter Schrenk, Jörg Fahrer

**Affiliations:** 1 Division of Food Chemistry and Toxicology, Department of Chemistry, RPTU Kaiserslautern-Landau, Erwin-Schroedinger-Str. 52, 67663 Kaiserslautern, Germany; 2 Division of Molecular Cell Biology, Department of Environment and Nature Science, Brandenburg University of Technology Cottbus-Senftenberg, 01968 Senftenberg, Germany

**Keywords:** Cytotoxicity, Genotoxicity, Benchmark dose modeling, Pyrrolizidine alkaloids, Primary human hepatocytes, Potency ranking, γH2AX, p53, DNA damage

## Abstract

Pyrrolizidine alkaloids (PAs) occur as contaminants in plant-based foods and herbal medicines. Following metabolic activation by cytochrome P450 (CYP) enzymes, PAs induce DNA damage, hepatotoxicity and can cause liver cancer in rodents. There is ample evidence that the chemical structure of PAs determines their toxicity. However, more quantitative genotoxicity data are required, particularly in primary human hepatocytes (PHH). Here, the genotoxicity of eleven structurally different PAs was investigated in human HepG2 liver cells with CYP3A4 overexpression and PHH using an in vitro test battery. Furthermore, the data were subject to benchmark dose (BMD) modeling to derive the genotoxic potency of individual PAs. The cytotoxicity was initially determined in HepG2-CYP3A4 cells, revealing a clear structure–toxicity relationship for the PAs. Importantly, experiments in PHH confirmed the structure-dependent toxicity and cytotoxic potency ranking of the tested PAs. The genotoxicity markers γH2AX and p53 as well as the alkaline Comet assay consistently demonstrated a structure-dependent genotoxicity of PAs in HepG2-CYP3A4 cells, correlating well with their cytotoxic potency. BMD modeling yielded BMD values in the range of 0.1–10 µM for most cyclic and open diesters, followed by the monoesters. While retrorsine showed the highest genotoxic potency, monocrotaline and lycopsamine displayed the lowest genotoxicity. Finally, experiments in PHH corroborated the genotoxic potency ranking, and revealed genotoxic effects even in the absence of detectable cytotoxicity. In conclusion, our findings strongly support the concept of grouping PAs into potency classes and help to pave the way for a broader acceptance of relative potency factors in risk assessment.

**Supplementary Information:**

The online version contains supplementary material available at 10.1007/s00204-023-03482-8.

## Introduction

Pyrrolizidine alkaloids (PAs) are toxins, which are formed in 3% of the worldwide flowering plants as a defence against herbivores (Chen et al. 2010). More than 660 individual PAs and *N*-oxide derivatives were identified in over 6000 plants, with *Asteraceae*, *Boraginaceae*, *Heliotropiaceae* and *Fabaceae* as major PA-producing plant families (Fu et al. 2004; Mattocks 1986; Yang et al. 2019). In the last decades, intoxications in lifestock and wildlife were reported due to the consumption of PA producing plants and in humans by consumption of PA-contaminated foods (e.g., contaminated herbal teas or wheat flours) (Tamariz et al. 2018; Wiedenfeld and Edgar 2010). PA-contaminated food led to several outbreaks of acute liver disease in countries of the Global South like Afghanistan, South Africa and Tajikistan, characterized by hepatomegaly, ascites and liver failure, which are known symptoms for acute and sub-acute PA intoxications (Moreira et al. 2018). Although chronic PA intoxications are not documented (Merz and Schrenk 2016), a recent study with mutational signature analyses in > 1,000 liver cancer genomes revealed a link between PA exposure and liver cancer prevalent in Asian countries (He et al. 2021b).

At the chemical level, PAs are heterocyclic structures composed of a pyrrolizidine backbone (necine base) and one or two esterified acids (necine acids). Necine acids can be mono- or dicarboxylic acids, which show high structural diversity and differ in the length of the carbon chain, acidities and branching degrees (Wiedenfeld et al. 2008). Despite their high structural diversity, PAs can be classified according to the necin-base type and degree of esterification. There are four known necine base types, namely retronecine-, heliotridine-, otonecine- and platynecine-type. Only retronecine-, heliotridine- and otonecine-type PAs possess a double bond at the C1-C2 position (1,2-unsatured) within the necin base (Wiedenfeld et al. 2008). Depending on their degree of esterification, PAs can be classified as monoester, open diester or cyclic diester.

Only the 1,2-unsatured PAs are of toxicological concern, since they can undergo metabolic activation giving rise to genotoxic and potentially carcinogen species (Chen et al. 2010; Fu et al. 2004; Prakash et al. 1999). The liver is the primary target organ for PA toxicity, since the compounds are activated by hepatic cytochrome P450 (CYP) monooxygenases (mostly CYP3A, but also CYP2B subfamilies) (Edgar et al. 2015). This biotransformation results in the generation of reactive dehydro-pyrrolizidine derivatives (dehydro-PAs), which can quickly hydrolyse into another reactive intermediate ( ±)-6,7-dihydro-7-hydroxy-1-hydroxymethyl-5H-pyrrolizine (DHP). Both metabolites bind covalently to DNA and cellular proteins, thereby forming adducts and crosslinks (Edgar 2014; Fu 2017). Furthermore, PAs can induce gene mutations (e.g., in *p53* and *Kras*), sister chromatid exchange, chromosomal aberrations and micronuclei (Chen et al. 2010). In the liver, PAs not only damage hepatocytes, but also proximate liver sinusoidal endothelial cells (LSEC), which was attributable to the efflux of reactive metabolites from the hepatocytes and uptake into LSEC (Hessel-Pras et al. 2020). This has recently also been demonstrated using a co-culture model of HepG2 liver cells and metabolically deficient HeLa cells (Hadi et al. 2023). It is further noteworthy that PAs can also be transported to other organs such as the lungs, where they can cause toxicity (He et al. 2021a; Song et al. 2020).

PAs in food and herbal medicinal products are currently regulated as a sum parameter independent of their chemical structure and genotoxic potential. However, several studies were conducted in recent years using various liver cell models, which provided evidence for a clear structure–toxicity relationship (Allemang et al. 2018; Gao et al. 2020; Louisse et al. 2019; Rutz et al. 2020). The data strongly support the concept to classify PAs according to their genotoxic and cytotoxic potential as previously suggested (Merz and Schrenk 2016). Nevertheless, a systematic potency ranking in human liver models, particularly primary human hepatocytes, based on a comprehensive genotoxicity data set for benchmark dose (BMD) analysis is still missing.

Thus, we analyzed the cytotoxicity and genotoxicity of up to eleven structurally diverse PAs in human HepG2-CYP3A4 liver cells and in primary human hepatocytes (PHH). The cytotoxic potential was investigated by the resazurin reduction assay in both liver cell models and EC_50_ values were calculated to assess the relative cytotoxicity. The genotoxic potential was determined using the alkaline Comet assay as well as western blot analysis of the DNA damage markers γH2AX and p53 in HepG2-CYP3A4 cells and confocal immunofluorescence microscopy of γH2AX in PHH. Furthermore, the comprehensive data sets were subject to benchmark dose (BMD) modelling via PROAST to derive BMD values for assessing the relative genotoxic potential. Finally, the obtained data sets were used for potency ranking in HepG2-CYP3A4 cells vs. PHH.

## Results

### HepG2-CYP3A4 cells reveal clear impact of PA structure and cell proliferation on PA-triggered cytotoxicity

**Fig. 1:**
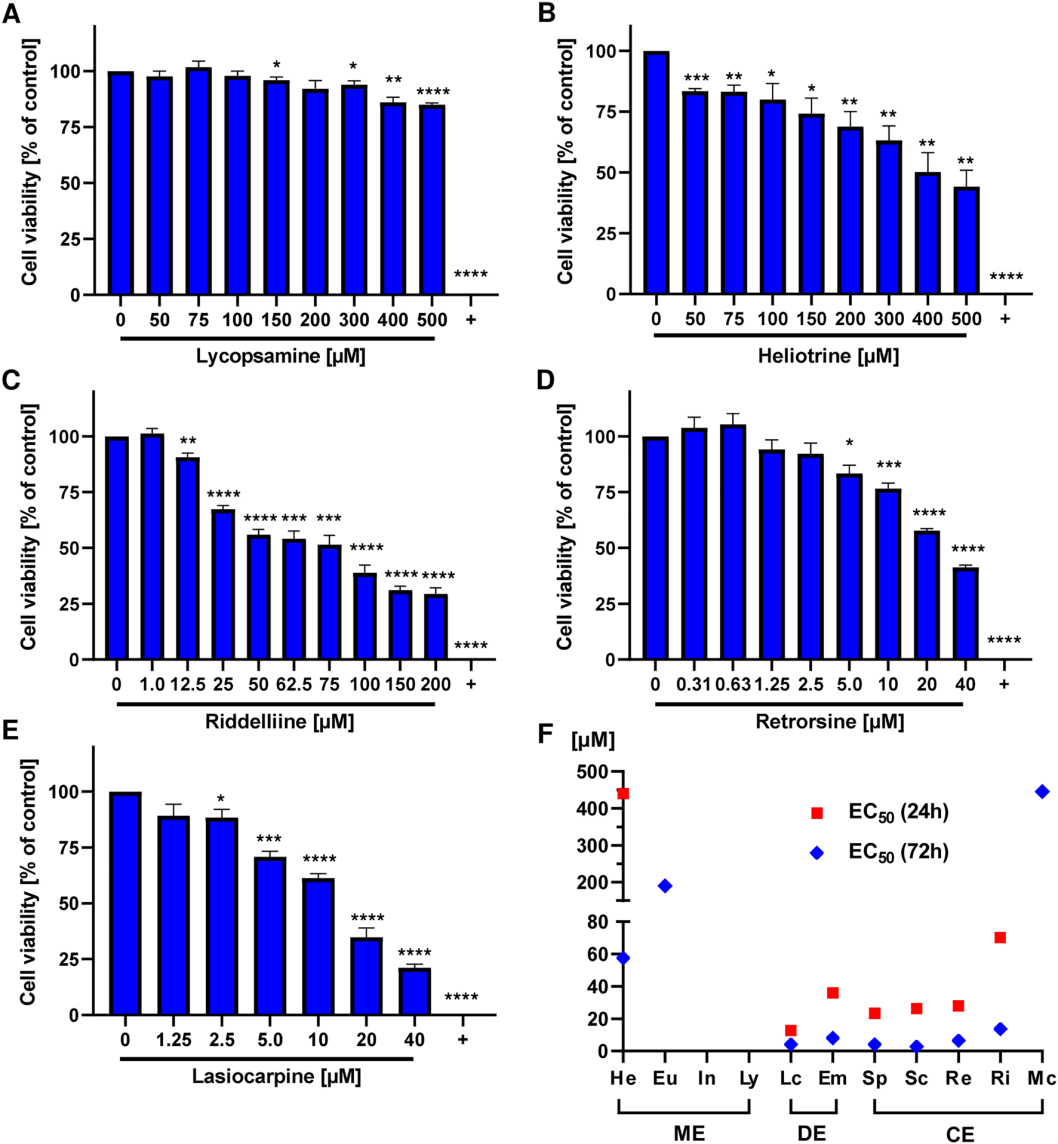
Cytotoxicity in HepG2-CYP3A4 cells triggered by structurally different PAs. A-E: Viability of HepG2-CYP3A4 cells 24 h after treatment with increasing concentrations of lycopsamine (**A**), heliotrine (**B**), riddelliine (**C**), retrorsine (**D**) and lasiocarpine (**E**). Saponin (0.1%) was used as a positive control (+) and solvent (0) as a negative control. Mean + SEM are shown for each incubation (*n* = 3, each measured as triplicates). Statistical analyses were performed using unpaired two-tailed Student’s t-test with respect to the negative control. *P ≤ 0.05, **P ≤ 0.01, ***P ≤ 0.001, ****P ≤ 0.0001. **F** EC_50_ values of HepG2-CYP3A4 after 24 and 72 h incubations with monoesters (ME), open diesters (DE), and cyclic diesters (CE). EC_50_ couldn’t be determined for Eu, In, Ly, and Mc, because the cytotoxic effects weren’t strong enough. *He* Heliotrine, *Eu* Europine, *In* Indicine, *Ly* Lycopsamine, *Lc* Lasiocarpine, *Em* Echimidine, *Sp* Seneciphylline, *Sc* Senecionine, *Re* Retrorsine, *Ri* Riddelliine, *Mc* Monocrotaline

First, we determined the cytotoxicity of eleven different PAs, representative for PA monoesters, cyclic diesters and open diesters (SI Fig. 1), using genetically engineered HepG2 cells with CYP3A4 overexpression. In these cells, CYP3A4 enzyme activity is comparable to that of PHH as shown previously (Herzog et al. 2015). HepG2-CYP3A4 cells were exposed to increasing PA concentrations (0 µM up to 500 µM, depending of the PA) for 24 h and cell viability was assessed by the resazurin reduction assay. All experiments included appropriate solvent controls as negative control and 0.1% saponine as technical positive control. No or only a slight decrease in cell viability was observed for the monoesters indicine (92%), lycopsamine (85%) and europine (77%) (Fig. 1A and SI Fig. 2A, B), whereas a strong decrease was seen for the monoester heliotrine (44%) in HepG2-CYP3A4 cells (Fig. 1B). The open diester echimidine and the cyclic diesters senecionine, retrorsine and riddelliine showed stronger cytotoxicity than all monoesters (Si Fig. 2C, D, Fig. 1C, D). Lasiocarpine and seneciphylline exerted the highest cytotoxicity with a decrease in cell viability to 21% at 40 µM and 24% at 100 µM, respectively (Fig. 1E and SI Fig. 2E). Interestingly, the cyclic diester monocrotaline was an exception among the tested cyclic diesters and caused only weak cytotoxicity at a concentration of 500 µM (77%) (SI Fig. 2F).

Next, we determined the relative cytotoxicity of each PA by calculating the effective concentration at 50% cell viability (EC_50_) from the concentration–response data, which was used to rank the PAs according to their cytotoxic potential (SI Fig. 3). The EC_50_ values of each PA after 24 h exposure in HepG2-CYP3A4 cells are shown in Fig. 1F. It should be noted that EC_50_ values could not be determined for europine, indicine, lycopsamine and monocrotaline due to their weak cytotoxicity. EC_50_ values between 10 and 70 µM were calculated for lasiocarpine, echimidine, seneciphylline, senecionine, retrorsine and riddelliine, whereby no striking differences in the cytotoxicity between cyclic and open diesters were observed. Furthermore, lasiocarpine displayed the highest cytotoxic potential with an EC_50_ of 12.6 µM in HepG2-CYP3A4 cells after 24 h exposure, followed by seneciphylline with an EC_50_ of 26.2 µM.

Subsequently, we wished to know how longer exposure might affect the cytotoxic potential of PAs in HepG2-CYP3A4 cells. To this end, cells were challenged with increasing concentrations (0 µM up to 500 µM, depending of the PA) for 72 h. Generally, we observed a higher concentration-dependent cytotoxicity in HepG2-CYP3A4 cells as compared to the shorter incubation period of 24 h. A moderate concentration-dependent decrease in cell viability was observed for lycopsamine and indicine, while a stronger drop in viability was seen for europine and heliotrine after 72 h exposure (SI Fig. 4A–D). Both lasiocarpine and senecionine showed the highest cytotoxicity after 72 h in HepG2-CYP3A4 cells (Fig. S4E and F), followed by the other diesters (SI Fig. 4G and H and SI Fig. 5A and B). Once again, the cytotoxicity of monocrotaline was comparable with the other tested monoesters, but not with the open or cyclic diesters (SI Fig. 5C). The EC_50_ values of the PAs were then calculated from the concentration–response data obtained in HepG2-CYP3A4 with 72 h PA exposure (SI Fig. 6). The EC_50_ values were consistently lower after 72 h as compared to 24 h (Fig. 1F). Indicine and lycopsamine showed the lowest cytotoxic potential and EC_50_ values could not be determined even after 72 h exposure. However, we were able to calculate the EC_50_ values of europine and monocrotaline after 72 h, which ranged between 200 and 500 µM. The other EC_50_ values were between 2 and 60 µM, including senecionine, seneciphylline, lasiocarpine, retrorsine, echimidine, riddelliine and heliotrine.

In summary, the panel of eleven representative PAs revealed a clear structure-dependent cytotoxicity and the following rank order with decreasing cytotoxic potential: lasiocarpine > seneciphylline = senecionine = retrorsine > echimidine > riddelliine >  > heliotrine >  > europine > monocrotaline > indicine = lycopsamine. In addition, our findings demonstrate that longer exposure enhances the cytotoxic effects induced by PAs in HepG2-CYP3A4 cells.

### Studies in PHH confirm the structure–dependent PA toxicity and potency ranking obtained in HepG2-CYP3A4 cells

**Fig. 2:**
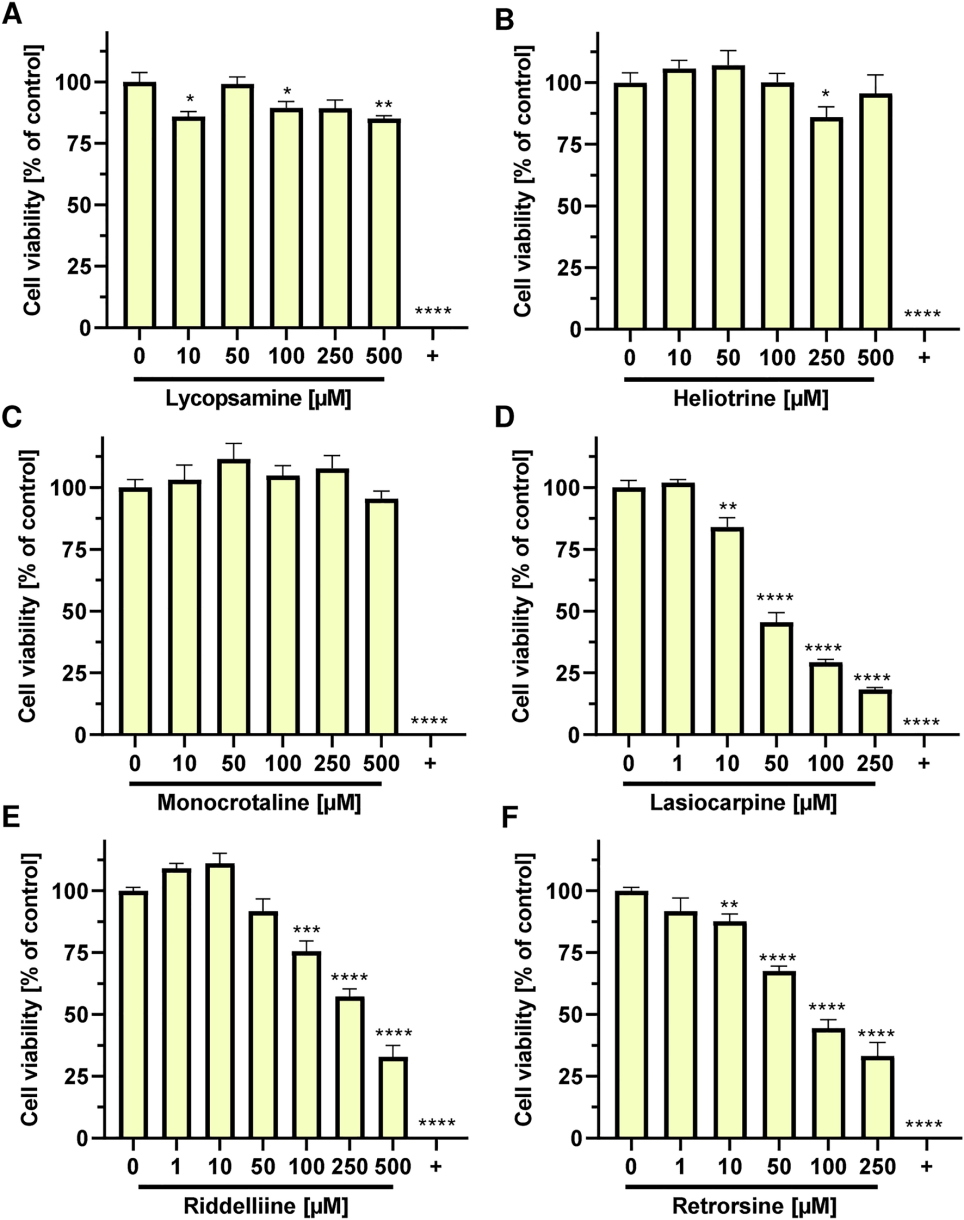
Structure-dependent cytotoxic effects of PAs in primary human hepatocytes. Viability of primary human hepatocytes after 24 h incubation with lycopsamine (**A**), heliotrine (**B**), monocrotaline (**C**), lasiocarpine (**D**), riddelliine (**E**), retrorsine (**F**). Saponin (0.1%) was used as a positive control (+) and solvent (0) as a negative control. Mean + SEM are shown (pooled hepatocytes from 5 donors, *n* = 2, each performed as triplicate). Statistical analyses were performed using unpaired two-tailed Student’s t-test with respect to the negative control. *P ≤ 0.05, **P ≤ 0.01, ***P ≤ 0.001, ****P ≤ 0.0001. *He* Heliotrine, *Ly* Lycopsamine, *Lc* Lasiocarpine; *Re* Retrorsine, *Ri* Riddelliine, *Mc* Monocrotaline

PHH are the gold standard in toxicological and pharmacological studies to investigate ADME profile and hepatotoxic effects of substances (Fraczek et al. 2013; Ruoss et al. 2020). PHH display full metabolic competence, express numerous influx and efflux transporters and exhibit no mutations in critical tumor suppressor genes or proto-oncogenes (Fraczek et al. 2013; Ruoss et al. 2020). Therefore, we were eager to analyze the cytotoxicity of PAs in PHH. The tested PAs were selected according to their relevance as contaminants in herbal medicinal products, different structure types and existing toxicological datasets in other cell models. In all assays, solvent served as negative control and 0.1% saponine as positive control. PHH were exposed to increasing PA concentrations (0 µM–500 µM, depending on the PA) for 24 h. The cytotoxicity was measured as described before and the relative potency of each PA was determined by calculating the EC_50_ from the concentration–response data. The results showed similar weak cytotoxic effects of heliotrine, lycopsamine and monocrotaline, with a cell viability of above 75% at 500 µM (Fig. 2A–C). Due to the low cytotoxicity of these PAs, it was not possible to calculate an EC_50_. In contrast to that, a significant dose-dependent decrease in cell viability was observed after treatment of PHH with lasiocarpine, retrorsine and riddelliine, with cell viabilities below 35% at the highest concentrations tested (Fig. 2D–F). Lasiocarpine showed the strongest cytotoxic effect with an EC_50_ of 45 µM, followed by retrorsine with a twofold higher EC_50_ value (98 µM) and riddelliine with a sevenfold higher EC_50_ value (292 µM) (SI Fig. 7A–C). The findings in PHH were supported by phase contrast microscopy, whereby higher concentrations led to decreased cell density, cell detachment and morphological changes (SI Fig. 8A-D). Altogether, the panel of six representative PAs showed a clear structure-dependent cytotoxicity and the following rank order with decreasing cytotoxic potential: lasiocarpine > retrorsine > riddelliine >  > heliotrine = monocrotaline = lycopsamine.

### Structure-dependent genotoxicity of different PAs shown by an in vitro genotoxicity test battery in HepG2-CYP3A4 cells

**Fig. 3:**
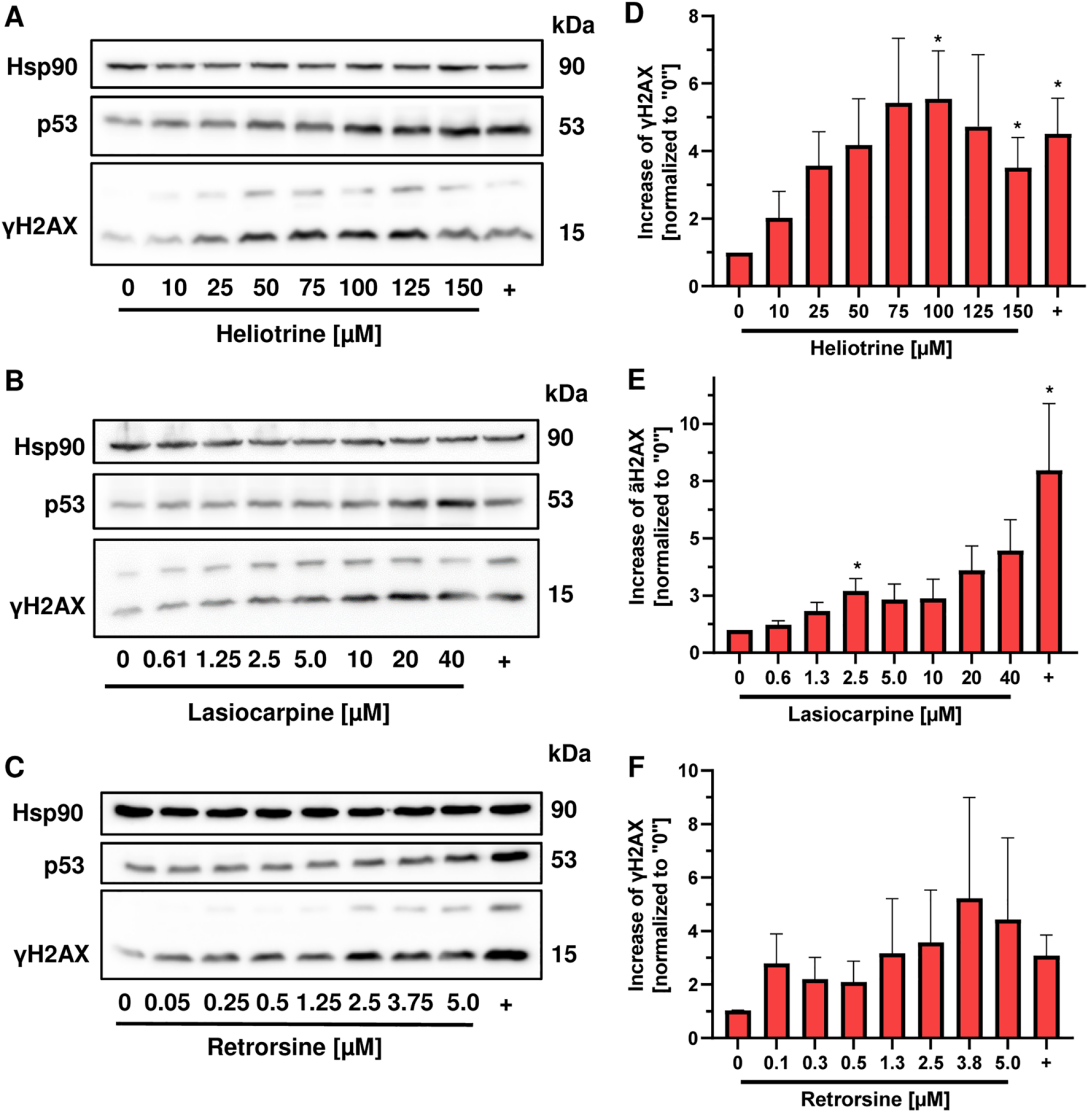
Structure-dependent γH2AX and p53 formation in HepG2-CYP3A4 cells triggered by different PAs. A-C: Representative western blots of γH2AX and p53 after 24 h incubation with heliotrine (**A**), lasiocarpine (**B**) and retrorsine (**C**) in HepG2-CYP3A4 cells. Etoposid was used as a positive (+) and solvent as a negative control (0). **D–F**: Densitometric evaluations of γH2AX after 24 h incubation with heliotrine (**D**), lasiocarpine (**E**) and retrorsine (**F**) in HepG2-CYP3A4. Hsp90 served as loading control. γH2AX level relative to the loading control and normalized versus the negative control. Mean + SEM of three independent experiments (*n* = 3). Statistical analyses were performed using unpaired two-tailed Student’s t-test with respect to the negative control. *P ≤ 0.05, **P ≤ 0.01, ***P ≤ 0.001, ****P ≤ 0.0001

Next, we investigated the impact of the PA structure on genotoxicity in HepG2-CYP3A4 cells using the established DNA damage markers γH2AX and p53 (Fahrer et al. 2015; Nikolova et al. 2014). The histone variant H2AX is phosphorylated (γH2AX) in response to DNA double-strand breaks and replication stress by apical DNA damage response (DDR) kinases (Kinner et al. 2008). The tumor suppressor protein p53 is known to be activated by genotoxic stress, resulting in its accumulation and p53-dependent regulation of downstream pathways such as DNA repair, cell cycle and cell death (Brady and Attardi 2010). HepG2-CYP3A4 cells were incubated with increasing PA concentrations for 24 h. The PA concentrations were chosen according to the determined EC_50_ values, with the highest concentration not exceeding the EC_50_ value. Solvent treated cells were used as negative control and the anticancer drug etoposide (10 µM) was included as positive control. Retrorsine induced a more than twofold induction of γH2AX already at the lowest tested concentration of 0.05 µM, whereas lasiocarpine and heliotrine showed a more than twofold increase in γH2AX at 1.3 µM and 10 µM, respectively (Fig. 3A–F). Comparable genotoxic effects were seen for p53 accumulation (SI Fig. 9A–C). A structure-dependent genotoxicity was also demonstrated for other PA congeners (SI Fig. 10–12). For example, seneciphyilline showed at 0.4 µM a significant increase of γH2AX (SI Fig. 10E and SI Fig. 12D), while a comparable effect was not observed below and at 100 µM europine (SI Fig. 10B and SI Fig. 11B). In general, most of the PAs showed a concentration-dependent increase in γH2AX and p53. However, lycopsamine (SI Fig. 11G and H) and monocrotaline (SI Fig. 12A and B) caused no clear concentration-related accumulation of both genotoxicity markers. Intriguingly, heliotrine provoked a more than twofold induction of both γH2AX and p53 at a concentration of 10 and 25 µM respectively, whereas the other monoesters europine and indicine showed the same effects at fivefold higher concentrations (50–150 µM) (SI Fig. 11A–D). The open diesters lasiocarpine and echimidine were equally genotoxic, with a twofold induction of γH2AX and p53 between 1.3 and 2.5 µM (Fig. 3B, SI Fig. 9B, SI Fig. 11E and F). With regard to the cyclic diesters, a wide concentration range between 0.05 and 12.5 µM depending on the individual PA was required for a twofold induction of the DNA damage markers (SI Fig. 11 and 12). Interestingly, the genotoxic effects of the open diesters were detected at the same concentration range as for cyclic diesters.

**Fig. 4:**
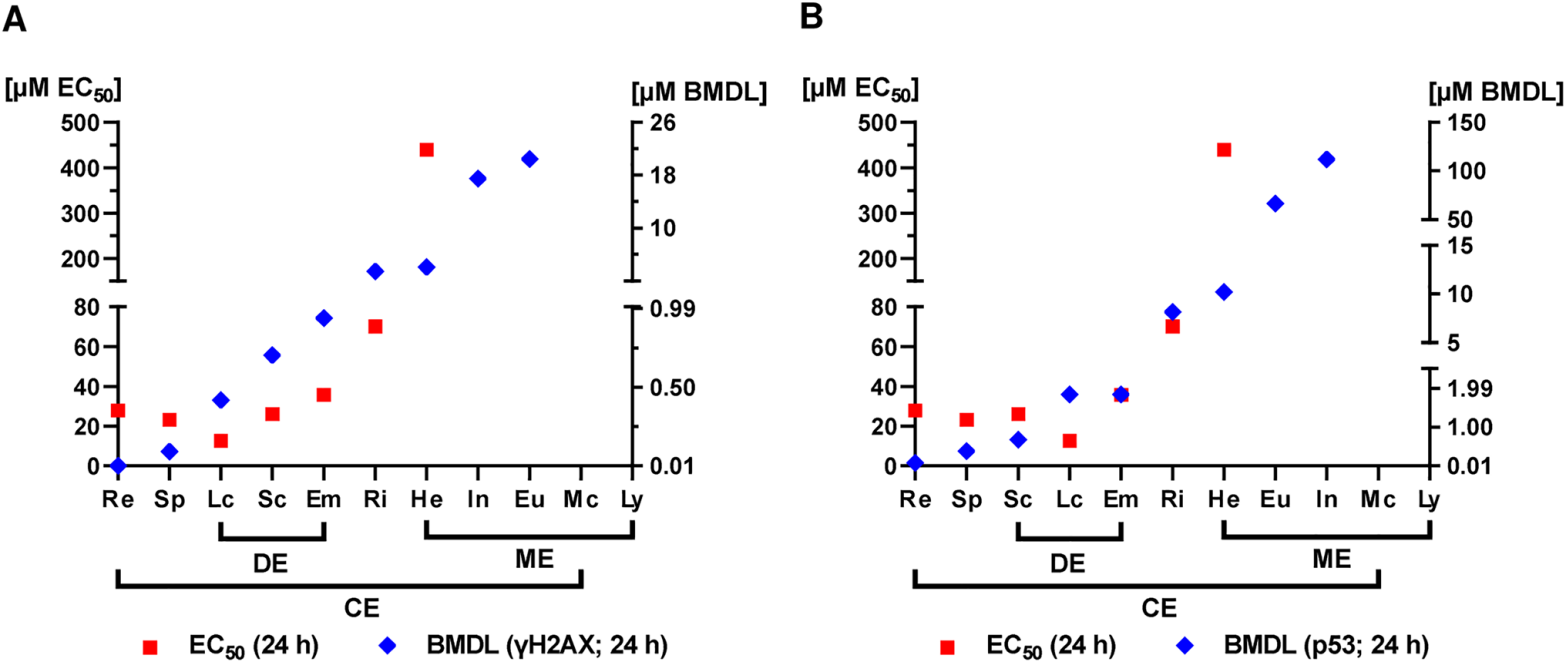
Genotoxic potency ranking of all tested PAs using the p53 and γH2AX data. A-B: BMDL values of γH2AX (**A**) and p53 (**B**) from western blot analysis and EC_50_ values of the 11 PAs including monoesters (Eu, He, In, Ly), open diesters (Em, Lc), and cyclic diesters (Mc, Re, Ri, Sc, Sp). BMDL values of the PAs depicted according to genotoxic potency. *He* Heliotrine, *Eu* Europine, *In* Indicine, *Ly* Lycopsamine, *Lc *Lasiocarpine, *Em* Echimidine, *Sp* Seneciphylline, *Sc* Senecionine, *Re* Retrorsine, *Ri* Riddelliine, *Mc* Monocrotaline

The concentration–response data were used for benchmark dose (BMD) modeling to derive relative genotoxic potencies (Fig. 4, SI Figs. 13 and 14). To this end, a critical effect size (CES) of one, i.e., a doubling of the measured γH2AX and p53 levels as compared to controls, was used as benchmark response with a confidence interval of 90%. Interestingly, the calculated critical effect dose (CED; represents BMD) and the respective critical effect dose lower boundary (CEDL, represents the BMDL) values of γH2AX and p53 were almost in the same concentration range for most of the PAs (Tables S1 and S2; SI Fig. 15). Moreover, the BMDL values of the cyclic diesters and open diesters were all below 1 µM, except for riddelliine (Tables S1 and S2). Retrorsine showed the strongest genotoxic potency with a BMDL of 0.01 µM, which is between 42- and 93-fold lower compared to the BMDL values of the open diesters and 409 and 2050-fold lower as those of monoesters (SI Fig. 15, Tab. S1 and S2). BMD modeling for lycopsamine and monocrotaline could not be performed due to lack of a significant genotoxic response. Taken together, the individual genotoxic potency of the tested PAs was shown to be structure dependent and correlated very well with their cytotoxic potency (Fig. 4A, B). BMD modeling of the γH2AX and p53 data resulted in the following rank order with decreasing genotoxicity: retrorsine >  > seneciphylline > lasiocarpine > senecionine = echimidine > riddelliine = heliotrine > indicine > europine > lycopsamine = monocrotaline.

**Fig. 5:**
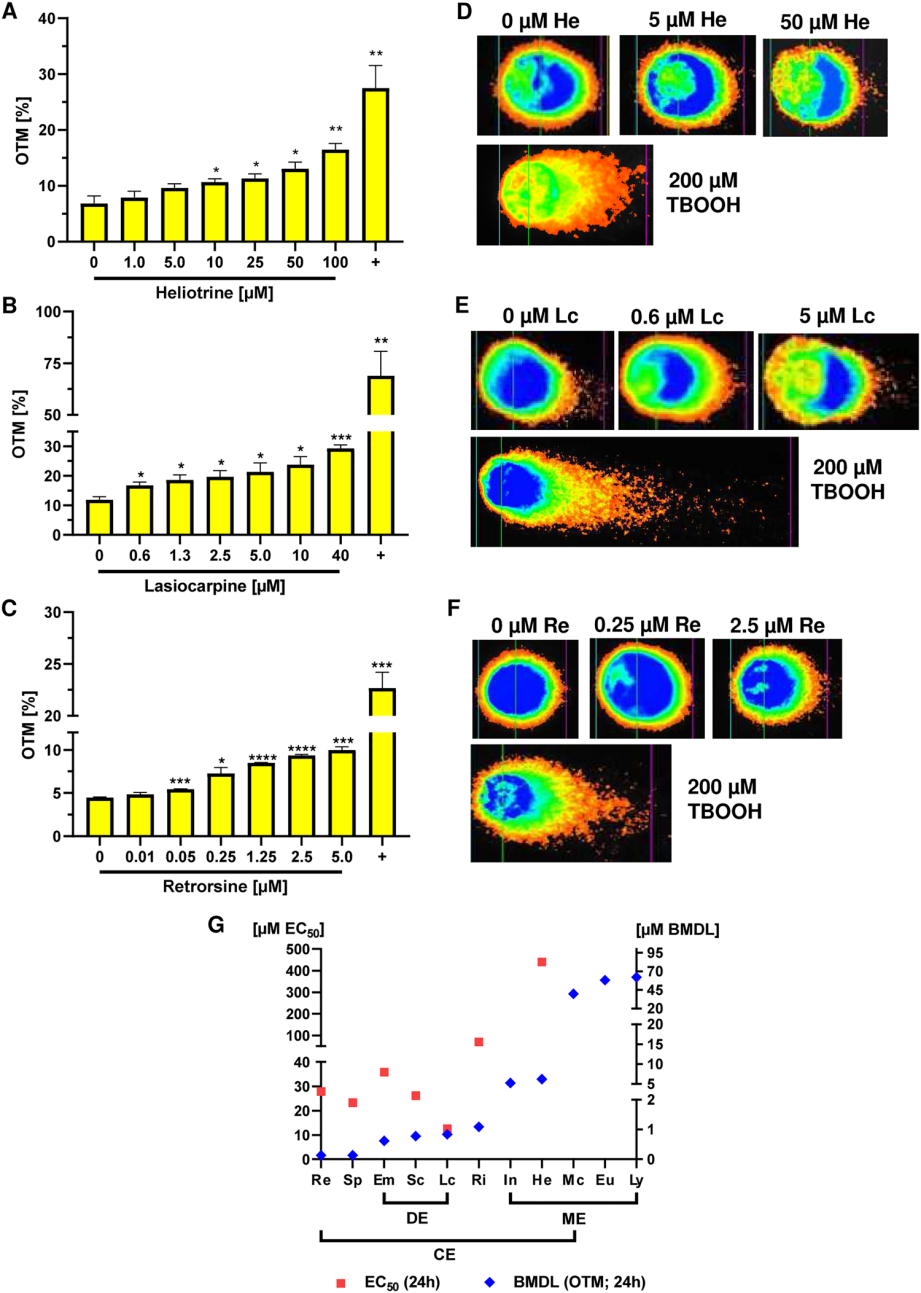
Structure-dependent DNA strand break formation in HepG2-CYP3A4 cells caused by different PAs. **A**–**C** Olive tail moments (OTM), which represents DNA strand break formation, in HepG2-CYP3A4 cells after 24 h incubation with heliotrine (**A**), lasiocarpine (**B**) and retrorsine (**C**). tBOOH was used as a positive (+) and solvent as a negative control (0). The alkaline Comet assay was performed as described. Mean + SEM are depicted (*n* = 3, except for heliotrine and europine *n* = 4). At least 50 comets per slide were counted and OTM was calculated by the software Comet Assay IV. Statistical analyses were performed using unpaired two-tailed Students t-test with respect to the negative control. *P ≤ 0.05, **P ≤ 0.01, ***P ≤ 0.001, ****P ≤ 0.0001. **D**–**F** Representative images of comets. **G** BMDL values derived from Comet data and EC_50_ values of the 11 PAs including monoester (Eu, He, In, Ly), open diester (Em, Lc), and cyclic diester (Mc, Re, Ri, Sc, Sp). BMDL values of the PAs shown according to genotoxic potency. *He* Heliotrine, *Eu* Europine, *In* Indicine, *Ly* Lycopsamine, *Lc* Lasiocarpine, *Em* Echimidine, *Sp* Seneciphylline, *Sc* Senecionine, *Re* Retrorsine, *Ri* Riddelliine, *Mc* Monocrotaline

Furthermore, we performed the alkaline Comet assay as additional genotoxicity endpoint with the same set of PAs. The alkaline Comet assay detects DNA single-strand breaks and double-strand breaks as well as alkali labile sites (Azqueta and Collins 2013; Moller et al. 2020). Thus, HepG2-CYP3A4 cells were incubated with increasing concentrations of PAs for 24 h. The PA concentrations were selected according to the determined EC_50_ values and comparable to those used for the determination of the genotoxicity markers p53 and γH2AX. Solvent treated cells were used as negative control and 200 µM tBOOH was applied as positive control. In agreement with the p53 and γH2AX data, retrorsine was very potent and caused a statistically significant increase in the OTM already at 0.05 µM, whereas a significant increase in OTM was detected at 0.6 µM lasiocarpine and 10 µM heliotrine (Fig. 5A–F). Importantly, a significant, concentration-dependent increase in OTM was observed for all tested PAs, including lycopsamine and monocrotaline (Fig. 5 and SI Fig. 16). The comet assay data were used for BMD modeling to derive relative genotoxic potencies. To this end, a CES of 0.5, which corresponds to a 1.5-fold increase of OTM as compared to controls, was used as benchmark response with a confidence interval of 90%. The calculated BMDL values of the cyclic and open diesters were in a range of 0.14 and 40 µM, whereas the BMDL values of the monoesters were between 6.2 and 62 µM (SI Fig. 17 and Tab. S3). Retrorsine and seneciphylline were the most genotoxic PAs with BMDL values of 0.14 µM (SI Fig. 17 and Tab. 1). Both PAs showed a three- to fivefold higher genotoxic potency as compared with the open diesters and a 38 – 444-fold higher genotoxic potency as monoesters. Among those, heliotrine and indicine displayed the highest genotoxicity, with BMDL values being about tenfold higher than those of europine and lycopsamine. Interestingly, the BMDL value of monocrotaline was in the same range as those of europine and lycopsamine (SI Fig. 17 and Tab. S3). In summary, the structure-dependent genotoxicity of the PAs was confirmed by the alkaline Comet assay. The calculated BMDL values were in very good agreement with those determined with the p53 and γH2AX assay (SI Fig. 13 and 14 vs. SI Fig. 17; Tab. S1–S3), providing the following rank order with decreasing genotoxic potential: retrorsine = seneciphylline > echimidine > senecionine = lasiocarpine > riddelliine > indicine > heliotrine > monocrotaline > europine > lycopsamine.

### PHH corroborate the genotoxic potency ranking performed in HepG2-CYP3A4 cells and reveal genotoxic effects in the absence of detectable cytotoxicity

**Fig. 6:**
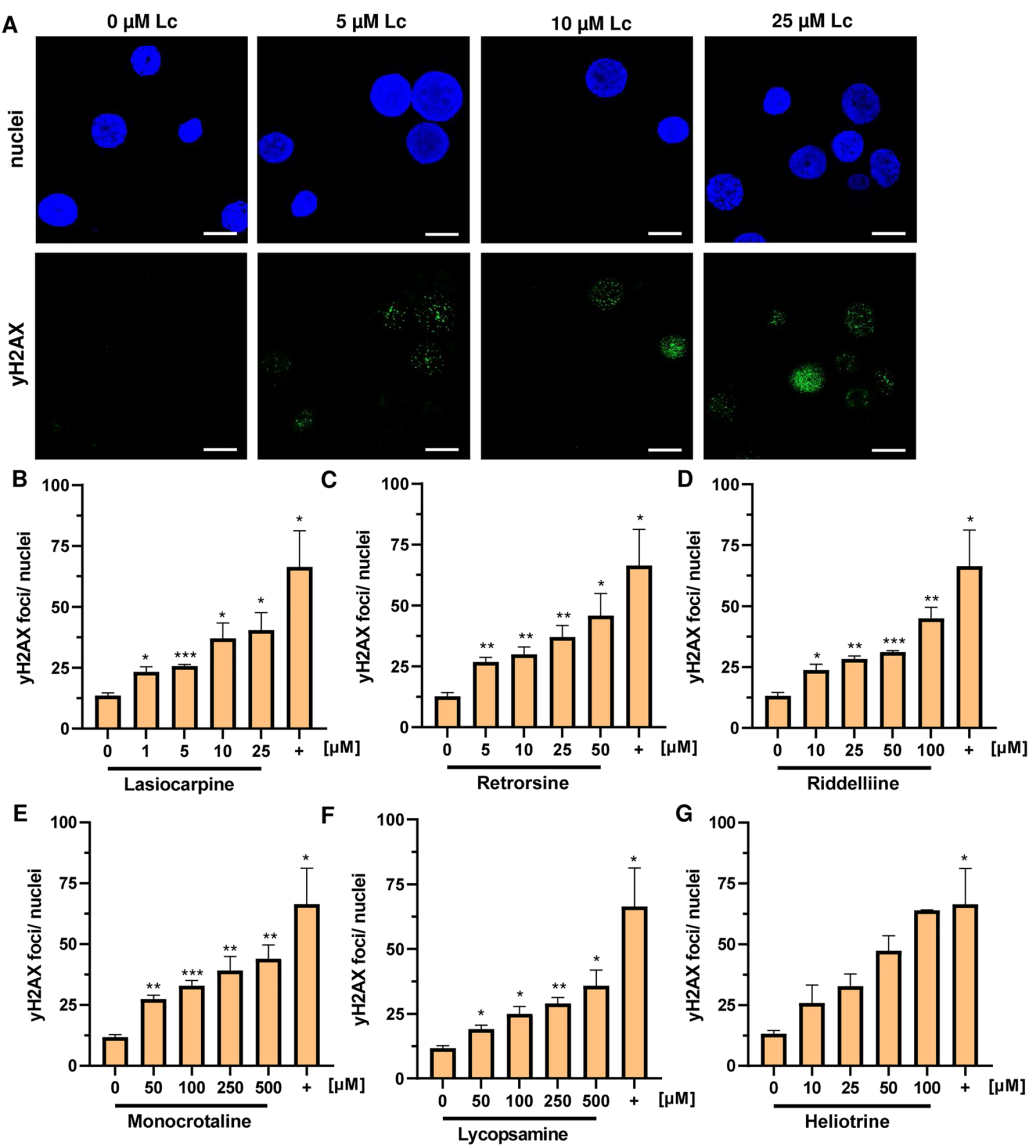
Formation of γH2AX foci in primary human hepatocytes by structurally different PAs. A: Representative images of primary human hepatocytes after 24 h incubation with 0–25 µM lasiocarpine. γH2AX was stained as described and detected by high resolution confocal microscopy (scale bar: 10 µm).** B**–**G** γH2AX foci in primary human hepatocytes after 24 h incubation with lasiocarpine (**B**), retrorsine (**C**), riddelliine (**D**), monocrotaline (**E**), lycopsamine (**F**) and heliotrine (**G**). *cis*-platin was served as positive control (+) and solvent as negative control (0). Mean + SEM are shown (pooled hepatocytes from 5 donors, *n* = 3), except for heliotrine (pooled hepatocytes from 5 donors, *n* = 2). At least 40 nuclei per sample were counted for γH2AX foci formation and evaluated with Zen 3.6 software. Statistical analyses were performed using unpaired two-tailed Students t-test with respect to the negative control. *P ≤ 0.05, **P ≤ 0.01, ***P ≤ 0.001, ****P ≤ 0.0001

Finally, we challenged PHH with increasing concentrations of the selected PAs lycopsamine, heliotrine, lasiocarpine, monocrotaline, retrorsine and riddelliine, which were also used in the cytotoxicity studies. Solvent treated cells served as negative control and 20 µM *cis-*platin was used as positive control. Importantly, every tested PA caused a concentration-dependent increase of γH2AX foci. PHH treated with 1 µM lasiocarpine showed on average 23 γH2AX foci per nucleus, whereas treatment with 5 µM retrorsine induced 27 γH2AX foci per nucleus (Fig. 6A–C). Twofold higher concentrations of heliotrine, fivefold higher concentrations of riddelline and 10- to 20-fold higher concentrations of monocrotaline and lycopsamine were required to cause the same γH2AX levels (Fig. 6D–G). It is noteworthy to mention that lycopsamine, although being weakly cytotoxic in PHH, induced γH2AX formation already at the lowest concentration tested, which was not observed in HepG2-CYP3A4 cells. Furthermore, monocrotaline was also genotoxic in PHH at the lowest concentration tested (50 µM), whereas in HepG2-CYP3A4 cells significant genotoxic effects were only detected at 200 µM using the alkaline Comet assay. Next, the relative genotoxicity of these PAs in PHH was determined by BMD modeling. To this end, a CES of 0.5, which corresponds to a 1.5-fold increase of γH2AX foci as compared to control, was used as benchmark response with a confidence interval of 90%. The calculated BMDL value of lasiocarpine was sixfold lower than the BMDL of retrorsine and 28-fold lower as compared to that of riddelline (SI Fig. 17 and Tab. S4). Heliotrine was as potent as riddelliine, but threefold more genotoxic than monocrotaline and even 15-fold more genotoxic than lycopsamine (SI Fig. 18 and Tab. S4). To summarize, all BMDL values were below 7 µM except for lycopsamine and were overall comparable with the results obtained in HepG2-CYP3A4, but with a six- to tenfold higher genotoxicity for monocrotaline and lasiocarpine in PHH. The following rank order with decreasing genotoxic potential was revealed in PHH: lasiocarpine > riddelliine > retrorsine > heliotrine > monocrotaline > lycopsamine.

## Discussion

**Fig. 7:**
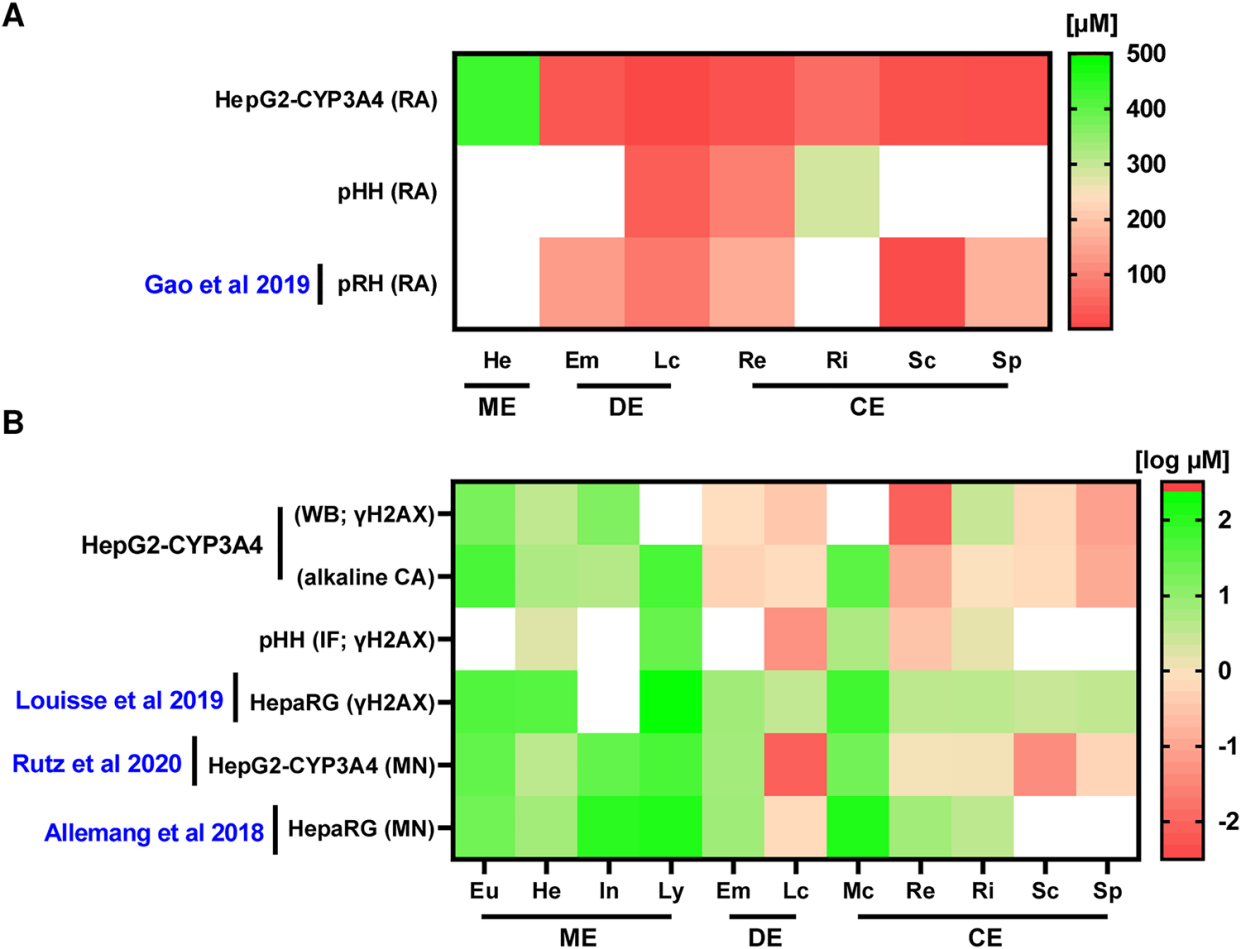
Comparison of BMD and EC_50_ values determined for structurally different PAs in various liver cell models. Comparison of cytotoxic (24 h) and genotoxic (24–48 h) potency of different PAs based on data in HepG2-CYP3A4, primary human hepatocytes (pHH), rat hepatocytes (pRH) as well as HepaRG cells. **A:** The cytotoxic effects of the PAs were measured by resazurin reduction assay. EC_50_ values were calculated to derive the relative cytotoxicity. Only PAs with existing EC_50_ value, i.e., sufficient cytotoxicity, are shown in the heatmap. Cytotoxicity is colour graded from high (dark red) to low (bright green). **B:** The genotoxic potentials were derived from γH2AX, Comet and micronuclei (MN) data, whereby data were transformed into BMDL values by PROAST. The BMDL values are shown in logarithmic concentrations [µM], i.e., a lower value represents a higher genotoxic potency. Data collected from the present work and published studies (Gao et al. 2020; Louisse et al. 2019; Allemang et al. 2018 and Rutz et al. 2020)

In this study, we investigated the structure-dependent toxicity of up to 11 PAs in human HepG2-CYP3A4 cells and PHH, which are a gold standard in pharmaco-toxicokinetic in vitro studies of the human liver. Importantly, we used an in vitro test battery consisting of three different genotoxicity endpoints that were combined with cytotoxicity testing side-by-side. Our findings in HepG2-CYP3A4 cells showed that cyclic and open diesters are highly cytotoxic already after 24 h, which is further potentiated after 72 h. These findings may be due to a higher sensitivity of proliferating cells and/or an accumulation of cell damage events over time. In contrast to that, monoesters with the exception of heliotrine exerted no cytotoxicity after 24 h incubation and were moderately cytotoxic after 72 h. The cytotoxic effects of the PAs were much higher than those reported in HepaRG cells, in which an EC_50_ calculation was not possible due to a lack of cytotoxicity (Glück et al. 2020). For example, lasiocarpine was the most cytotoxic PA in our cell model with an EC_50_ of 12 µM after 24 h. In HepaRG cells, a concentration of 250 µM reduced viability by 50% only (Glück et al. 2020), indicating a moderate sensitivity of HepaRG cells towards the cytotoxic effects of PAs. Furthermore, the PA-triggered cytotoxic effects were also stronger in PHH as compared to HepaRG cells, although PHH do not proliferate at all. Our study demonstrated that the ranking of PAs according to their cytotoxicity in PHH is comparable with that in HepG2-CYP3A4 cells, although the cytotoxic potency was generally lower in PHHs. An attenuated cytotoxicity was also observed in primary rat hepatocytes (pRH) (Gao et al. 2020). Most of the EC_50_ values in pRH were almost twofold higher as in PHH and between four- and ninefold higher than in HepG2-CYP3A4 (Fig. 7A). Intriguingly, a comparable rank order was observed for all tested PAs in both rodent and human liver cell models. The lower cytotoxicity in primary hepatocytes is most likely attributable to the lack of cell proliferation, but other processes like the expression of transporters, their extensive metabolic capacity and the effect of cryopreservation (in case of pHH) on CYP activity could also be relevant (Fraczek et al. 2013; Ruoss et al. 2020; Kammerer and Küpper 2018). Senecionine was an exception in this regard and showed a similar cytotoxicity in pRH and in HepG2-CYP3A4 (Fig. 7A). Interestingly, a recent study revealed an EC_50_ value of 26 µM for senecionine in primary mouse hepatocytes (pMH) (Hessel-Pras et al. 2020), which fits very well to the EC_50_ values for senecionine determined in HepG2-CYP3A4 and pRH.

Our genotoxicity test battery with the endpoints γH2AX, p53 and DNA strand break induction demonstrated a clear structure-dependent toxicity of the tested PAs in HepG2-CYP3A4 cells, which was consistent with the results obtained in PHH. The determined BMDL values revealed a higher genotoxic potency for diesters compared to monoesters, except for monocrotaline (Fig. 7B). Retrorsine showed the strongest genotoxic potential with a BMDL value of 0.01 µM followed by seneciphylline based on the γH2AX data (Fig. 7B). A similar pattern of γH2AX induction was obtained in another study conducted in HepaRG cells, which included PAs and PA N-oxides (Louisse et al. 2019). However, the BMDL values determined in HepaRG cells were 1.3- to 440-fold lower as compared to those found in HepG2-CYP3A4 cells (Fig. 7B). This might be explained by differences in cell proliferation, expression of influx/efflux transporters or other factors that influence the cellular sensitivity. Consistent with our findings in PHH, both human liver cell models provided comparatively high BMDL values for the diester monocrotaline, which was even higher than those of most monoesters. The weak genotoxic potency of monocrotaline might be due to lack of efficient bioactivation or other toxicokinetic parameters, such as hepatocyte uptake or efflux. Interestingly, monocrotaline was found to be mainly metabolized by rat liver S9 and only to a small extent by lung S9 fractions (Song et al. 2020). Nevertheless, the ratio of pyrrole-protein adducts in lung vs. liver was significantly higher for monocrotaline than that of other tested PAs, which might indicate a preferential lung toxicity of monocrotaline. A recent in vivo study confirmed that bioactivation of monocrotaline by liver CYPs and transport of the reactive metabolites via the blood stream is required for its lung toxicity (He et al. 2021a).

Furthermore, our study provided evidence that p53 is a valuable and sensitive genotoxicity marker for PAs, which allows BMD modeling to derive the genotoxic potency. The BMDL values based on the γH2AX and p53 data sets were almost equal among the tested open diesters and cyclic diesters, but differed to some extent within the monoesters. All monoesters showed a 2.5- to eightfold higher BMDL value with p53 as endpoint, which might indicate differences in the genotoxic mode of action of monoesters as compared to diesters. The observed concentration-dependent accumulation of p53 after treatment of HepG2-CYP3A4 cells with all 11 tested PAs is in line with transcriptome studies performed in the same cell model, which revealed an upregulation of the p53 pathway (Abdelfatah et al. 2022). An activation of the p53 signaling pathway in rat liver was also found in a 28-day feeding study (Ebmeyer et al. 2020), highlighting the relevance of p53 as genotoxicity marker for PAs.

As third genotoxicity endpoint, we investigated the PA triggered DNA damage using the alkaline Comet assay. The obtained BMDL values matched very well to those determined with the two other endpoints (i.e., yH2AX and p53). Moreover, we were able to derive BMDL values for the weakly genotoxic PAs lycopsamine and monocrotaline, highlighting the sensitivity of the Comet assay. PA-triggered DNA strand breaks can result in chromosomal damage and formation of micronuclei. This has also been investigated in a structure-dependent manner using metabolically competent HepG2-CYP3A4 cells and HepaRG cells (Rutz et al. 2020; Allemang et al. 2018) as well as HepG2 cells with CYP induction by rifampicin pretreatment (Hadi et al. 2021). The two studies in metabolically competent cells revealed BMDL values in the range of 0.01–8.5 µM for the tested cyclic and open diesters as well as heliotrine, whereas the BMDL values for monoesters and monocrotaline ranged between 25 and 154 µM (Fig. 7B) (Rutz et al. 2020; Allemang et al. 2018). These results are in good agreement with our findings obtained by a genotoxicity test battery, in particular the alkaline Comet assay (Fig. 7B).

Since our used HepG2 cell model was genetically engineered only for CYP3A4 expression, we conducted further genotoxicity experiments in PHH, which express the whole array of phase I and phase II enzymes (Fraczek et al. 2013). It is noteworthy that PAs are not exclusively metabolized via CYP3A4, but bioactivation also involves CYP3A5 (e.g., lasiocarpine) and CYP3A7 (e.g., riddelliine) to some extent as shown in a panel of genetically engineered TK6 cells (Li et al. 2020). Previous studies with recombinant human CYP supersomes revealed that the vast majority of tested PAs are substrates for CYP3A4 (Ruan et al. 2014). One exception was monocrotaline, which was predominantly metabolized via CYP2A6 (Ruan et al. 2014). This CYP isoform plays also an important role for the metabolism of retrorsine together with CYP3A4 (Ruan et al. 2014). In PHH, we analyzed nuclear γH2AX formation using high-resolution confocal microscopy, which requires less cells than the western blot analysis used in HepG2-CYP3A4 cells. Our findings showed that lasiocarpine and retrorsine were the most potent genotoxic PAs in PHH with BMDL values between 0.06 and 0.4 µM. Furthermore, the genotoxic potencies of the tested PAs in PHH were generally comparable with those obtained in HepG2-CYP3A4 cells, but revealed a six- to tenfold higher genotoxicity for monocrotaline and lasiocarpine in PHH. This may be attributable due to the presence of further CYP enzymes in PHH, such as CYP2A6 and CYP3A5, which contribute to the metabolic activation of those PAs as pointed out above. Interestingly, lasiocarpine followed by riddelliine and heliotrine caused the highest DHP-DNA adduct levels in a rat sandwich-culture hepatocytes (Lester et al. 2019), which is in line with our findings in PHH.

**Fig. 8:**
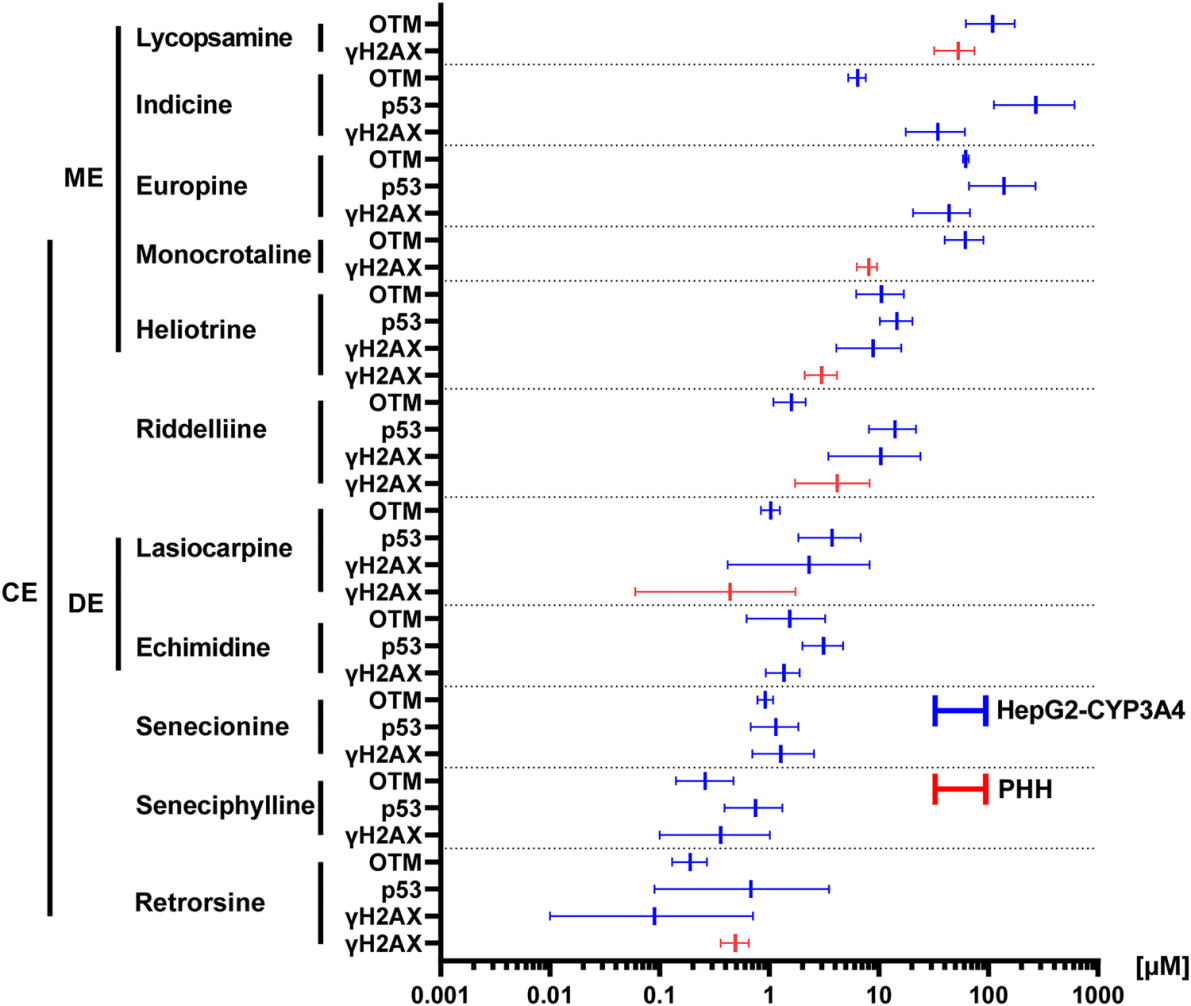
Comprehensive genotoxic potency ranking of PAs in HepG2-CYP3A4 cells and PHH. The concentration–response data for the endpoints γH2AX, p53 and Comet assay (OTM) were used to derive BMD values including the 90% confidence intervals (BMDL, BMDU) by PROAST. PAs were then ranked according to their genotoxic potency based on their mean of all derived BMD values (equally weighted) determined in HepG2-CYP3A4 cells. Depicted are the BMD confidence interval plots for the different endpoints in HepG2-CYP3A4 cells (blue) and PHH (red)

Finally, we made an integrated approach for potency ranking using all genotoxicity data and depicted the determined BMD values with the confidence intervals (BMDL and BMDU) to account for data variability. To this end, PAs were ranked according to their genotoxic potency based on the mean of the BMD values from all genotoxicity endpoints in HepG2-CYP3A4 cells (Fig. 8). Retrorsine showed the highest and lycopsamine the lowest genotoxic potency from all tested PAs, which was consistent with the rank order observed in PHH (Fig. 8). Interestingly, the BMD values of monocrotaline determined in HepG2-CYP3A4 and PHH were two- to 40-fold lower as compared to the BMD values of riddelliine, which is in good agreement with a previous study in HepaRG cells (Louisse et al. 2019).

In 2016, Merz and Schrenk introduced the concept of relative (toxicity) potency (REP) factors to group PAs with respect to their toxicity (Merz and Schrenk 2016). This was based on available data comprising acute toxicity in rodents, cytotoxicity in hepatocellular carcinoma cells and genotoxicity in *Drosophila*. In the meantime several in vitro studies have been performed in rodent and human liver cell models (Allemang et al. 2018; Gao et al. 2020; Louisse et al. 2019; Rutz et al. 2020), largely corroborating the suggested interim REPs within one order of magnitude (Schrenk et al. 2022). Our comprehensive analysis of different genotoxicity endpoints in human HepG2-CYP3A4 cells and PHH as gold standard in preclinical toxicity testing reinforced the existing database and provisional iREPs. Particularly, we were able to demonstrate the structure-dependent toxicity of PAs in PHH with a similar potency ranking as observed in metabolically competent liver cancer cell lines (i.e., HepG2-CYP3A4 and HepaRG). Furthermore, our study highlights that genotoxic effects in PHH occur already at PA concentrations that cause no cytotoxicity. Our data contribute to a broader database on cyto- and genotoxic effects and their concentration–response relationships for various PAs in mammalian cell culture. It is not surprising that the various tests did not and will not result in identical BMDs due to the complexity and diversity of the endpoints tested. Attempts are currently under way to harmonize the methodology for benchmark derivation between tests including data from other laboratories. Such a comprehensive inventory will allow carrying out a density distribution (probability) analysis over all available and relevant concentration–response relationships to derive the most consolidated (likely) REP factors. Altogether, this will help to pave the way for a broader acceptance of REPs in PA risk assessment.

## Material and methods

### Cell culture and treatment

HepG2-CYP3A4 cells were generated as previously described (Herzog et al. 2015). CYP3A4 expression was confirmed by western blot analysis (Data not shown). HepG2-CYP3A4 cells were maintained in DMEM high glucose supplemented with 10% FCS, 1% P/S and 3 µg/mL blasticidin S hydrochloride (Carl Roth, Karlsruhe, Germany). HepG2-CYP3A4 cells were cultured at 37 °C in humidified atmosphere of 5% CO_2_. Cell culture medium and supplements were obtained from Gibco Life Technologies (Darmstadt, Germany) and Pan Biotech (Aidenbach, Germany). Cell lines were mycoplasma negative, as demonstrated by routine PCR testing using Venor^®^GeM OneStep (Berlin, Germany). Cryopreserved primary human hepatocytes (PHH) pooled from five Caucasian donors were obtained from Thermo Fisher Scientific (Massachusetts, USA). The primary cells were thawed and plated according to the protocol provided by Invitrogen. All reagents were from Thermo Fisher. Cells were thawed at 37 °C in the water bath, transferred to 45 mL hepatocyte thaw media, centrifuged at 500 *g* for 5 min at room temperature (RT) and re-suspended in plating medium. The cell viability was assessed using trypan blue exclusion and cells with a viability of ≥ 90% were used for experiments. Before seeding, the wells were coated with 50 µg/ mL Collagen type I for at least 1 h (Herzog et al. 2016). Cells were cultured at 37 °C in humidified atmosphere of 5% CO_2_ for 6 h. Afterwards, the cells were attached and ready for exposure. The plating medium was replaced with incubation medium mixed with the test compounds and cells were treated for 24 h. Collagen type I (rat) was purchased from Corning (New York, USA) and sterile-filtered 50 µg/mL stock solution in 0.8 M acetic acid was prepared for coating.

### Compounds and treatment

The investigated PAs (indicine, heliotrine, lycopsamine, europine, echimidine, lasiocarpine, riddelliine, retrorsine, monocrotaline, senecionine, seneciphylline) were of highest purity and obtained from Phytolab (Vestenbergsgreuth, Germany). Stock solutions of PAs were prepared in suitable solvents (50–150 mM) and stored at −20 °C. Heliotrine, lycopsamine, echimidine, lasiocarpine, riddelliine, monocrotaline were dissolved in DMSO, whereas senecionine and seneciphylline were dissolved in a 1:1 mixture (v:v) of DMSO and acetonitrile. Retrorsine was dissolved in ethanol. Stock solutions were diluted in cell culture medium to reach final concentrations in the cell experiments.

### Analysis of cell viability and determination of EC_50_ values

HepG2-CYP3A4 cells (45,000 cells/ well) were seeded on 96-well plates, grown overnight and then treated with increasing PA concentrations. PHH were seeded on collagen type I coated 96-well plates at density of 62,500 cells/ well and treated as described above. Saponine (0.1%) served as positive control and solvent as negative control. Cell viability was determined as described previously (Carlsson et al. 2022). After 24 h and 72 h, cells were washed with PBS and incubated for 1 h with DMEM low glucose (-FCS; -P/S) or William’s E Medium (1X) supplemented with 10% 440 µM Resazurin-NaCl-Pi-solution (0,1% dimethylformamide; 1.1 mM KH_2_PO_4_, 154 mM NaCl, 3.7 mM Na_2_HPO_4_) at 37 °C in humidified atmosphere of 5% CO_2_. Cell viability was measured using a microplate reader (Fluoroskan HSCENT FL, ThermoScientific and Spark®, Tecan) at 544 nm for absorbance and 590 nm for emission. The effective concentration, at which cell viability was reduced by 50% (EC_50_), was calculated by semi-logarithmic transformation of the data and fitting with sigmoidal, non-linear curve using GraphPad Prims software (Version 9).

### SDS-PAGE and western blot analysis

HepG2-CYP3A4 cells (500,000 cells/ plate) were seeded on 3.5 cm plates, grown overnight and treated with increasing PA concentrations for 24 h. Cells were directly harvested with 1 × Laemmli loading buffer as described (Fahrer et al. 2014). Western blot analysis was then conducted as reported (Fahrer et al. 2013). Briefly, proteins were separated by SDS-PAGE and transferred onto a nitrocellulose membrane (PerkinElmer, Rodgau, Germany) with wet blot technique. The membrane was blocked with 5% nonfat dry milk in Tris-buffered saline (TBS)/ 0.1% Tween-20 (TBS-T) for 1 h at RT. Afterwards, primary antibody incubation was started overnight at 4 °C. The membranes were washed three times with TBS-T and incubated with appropriate secondary antibodies coupled with horseradish peroxidase for 1 h at RT. After additional washing steps, the membranes were incubated with Western Lighting® Plus-ECL (PerkinElmer, Rodgau, Germany) and proteins detected using a c300 chemiluminescence imager (Azure biosystems, Dublin, CA, USA). The primary antibodies directed against Hsp90 (F8, sc-13119) p53 (DO-1, sc-126) were obtained from Santa Cruz Biotechnology (Heidelberg, Germany). The primary antibody directed against γH2AX (phospho S139, ab81299), was from Abcam (Cambridge, UK). Secondary antibodies conjugated with horseradish peroxidase were purchased from Santa Cruz Biotechnology (sc-516102, Heidelberg, Germany) and Cell Signaling Technology (#7074, Danvers, Massachusetts, USA).

### Alkaline comet-assay

HepG2-CYP3A4 cells (500,000 cells/ plate) were seeded on 3.5 cm plates and exposed to PAs for 24 h. tBOOH (200 µM, 1 h) was used as positive control and solvent as negative control. The alkaline Comet assay was essentially performed as described (Dörsam et al. 2018), with slight modifications. Cell lysis was conducted for 60 min, followed by 20 min denaturation and electrophoresis for 30 min. The slides were stored under light exclusion before DNA staining with propidium iodide. At least 50 comets per slide were scored using an Axioskop 2 fluorescence microscope (Zeiss, Jena, Germany) and Comet Assay IV software (Perceptive Instruments, Bury St Edmunds, UK).

### Confocal immunofluorescence microscopy of γH2AX in PHH

PHH were seeded on collagen type I coated 12-well chamber slides at density of 10,000 cells/ well. The 12-well chamber slides were obtained from Ibidi^®^ (Gräfelfing, Germany) and used according to the manufacturer`s protocol. Cells were exposed to different concentrations of PAs (heliotrine, lasiocarpine, lycopsamine, monocrotaline, retrorsine and riddelliine) for 24 h. C*is*-platin were used as positive control and solvent as negative control. γH2AX immunofluorescence staining was essentially performed as described (Mimmler et al. 2016). After fixation with methanol for 10 min at − 20 °C, cells were washed with PBS and blocked with 5% BSA in PBS/ 0.3% Triton X-100 for 1 h at RT. Afterwards, incubation with primary antibody directed against γH2AX (1:1000 in PBS/ 0.3% Triton X-100; Millipore, Burlington, Massachusetts, USA) was performed overnight at 4 °C. Cells were washed with PBS and PBS/ 0.4 M NaCl and subsequently incubated with a secondary antibody labeled with Alexa Fluor 488 (1:400 in PBS/ 0.3% Triton X-100) for 1 h at RT. Cells were washed as mentioned before and embedded with Vectashield^®^ (Vector Labs, Burlingame, CA, USA). The samples were then analyzed using a Zeiss Axio Observer 7 microscope equipped with a 63x oil objective (plan-apochromat 63x/1.40 DIC M27) and LSM 900 confocal laser scanner (Carl Zeiss Microscopy, Jena, Germany). At least 40 nuclei per sample were analyzed for γH2AX foci by Zen 3.6 software (Carl Zeiss Microscopy, Jena, Germany). High resolution images were obtained using the Zeiss Airyscan.

### Determination of relative genotoxicity by Benchmark dose modelling

To derive the relative genotoxic potencies based on the western blot, Comet assay and immunofluorescence data, a free software available from the European Food and Safety Agency (EFSA), called PROAST, was used. Hereby, concentration–response relationships were modelled to determine the benchmark dose (BMD) at a defined benchmark response (BMR) and its related 90% lower and upper confidence limits (BMDL and BMDU). The software determined best fits out of different algorithms and the best four fits were summarized as mean as long as they fulfilled the requirements (lowest AIC value). The BMR were chosen according to the outcome of data, so that a higher threshold was set for the more semi-quantitative western blot data. A lower BMR for comet assay and immunofluorescence data were chosen, because 50 comets or > 40 cells per sample in each biological replicate were evaluated. Therefore, a 2.0-fold increase of γH2AX intensity from western blot data (BMD_100_, CES = 1.0) and 1.5-fold increase (BMD_50_; CES = 0.5) of OTM and γH2AX foci per nuclei were used as a benchmark response (CES, critical effect size). The means of BMDL and BMDU were calculated by averaging the four models.

### Statistical analysis

All experiments were performed independently at least three times, except otherwise stated. Results are presented as means ± standard error of the means (SEM) from representative experiments. Statistics were carried out by Graphpad Prism software (Version 9), statistical significance was defined as *P* < 0.05 and statistical analyses were performed using unpaired two-tailed Student’s t-test with respect to the negative control.

## Supplementary Information

Below is the link to the electronic supplementary material.

Supplementary file1 (PDF 2718 KB)

## Data Availability

All datasets generated and analyzed during this study were included in the manuscript and the supplementary information. They are also available from the corresponding author upon reasonable request.
