# Supplementary material for: Potency ranking of pyrrolizidine alkaloids in metabolically competent human liver cancer cells and primary human hepatocytes using a genotoxicity test battery
Source: Arch Toxicol. 2023 Mar 16;97(5):1413–28. doi: 10.1007/s00204-023-03482-8 (PMC10110667; doi:10.1007/s00204-023-03482-8)
Supplement: Supplementary file 1 — Supplementary file1 (PDF 2718 KB) [file 204_2023_3482_MOESM1_ESM.pdf]

Supplementary Figures

Figure S1

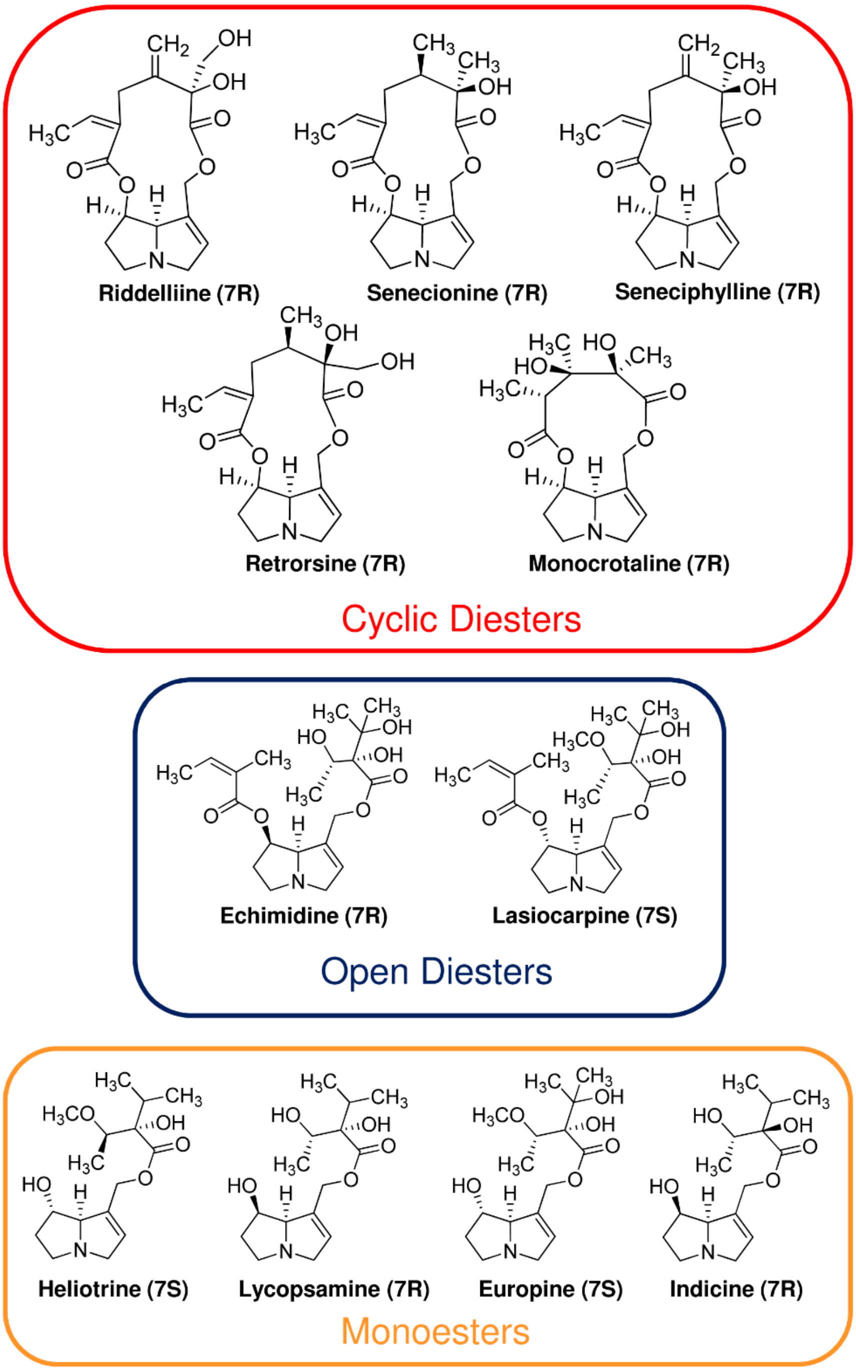

Figure S1: Chemical structures of the eleven PAs tested in human liver cells. As cyclic di-esters monocrotaline (7R), retrorsine (7R), riddelliine (7R), senecionine (7R) and seneciphylline (7R) were selected. Echimidine (7R) and lasiocarpine (7S) were chosen as open di-esters. Four mono-esters including europine (7S), heliotrine (7S), indicine (7R) and lycopsamine (7R) were tested.

Figure S2

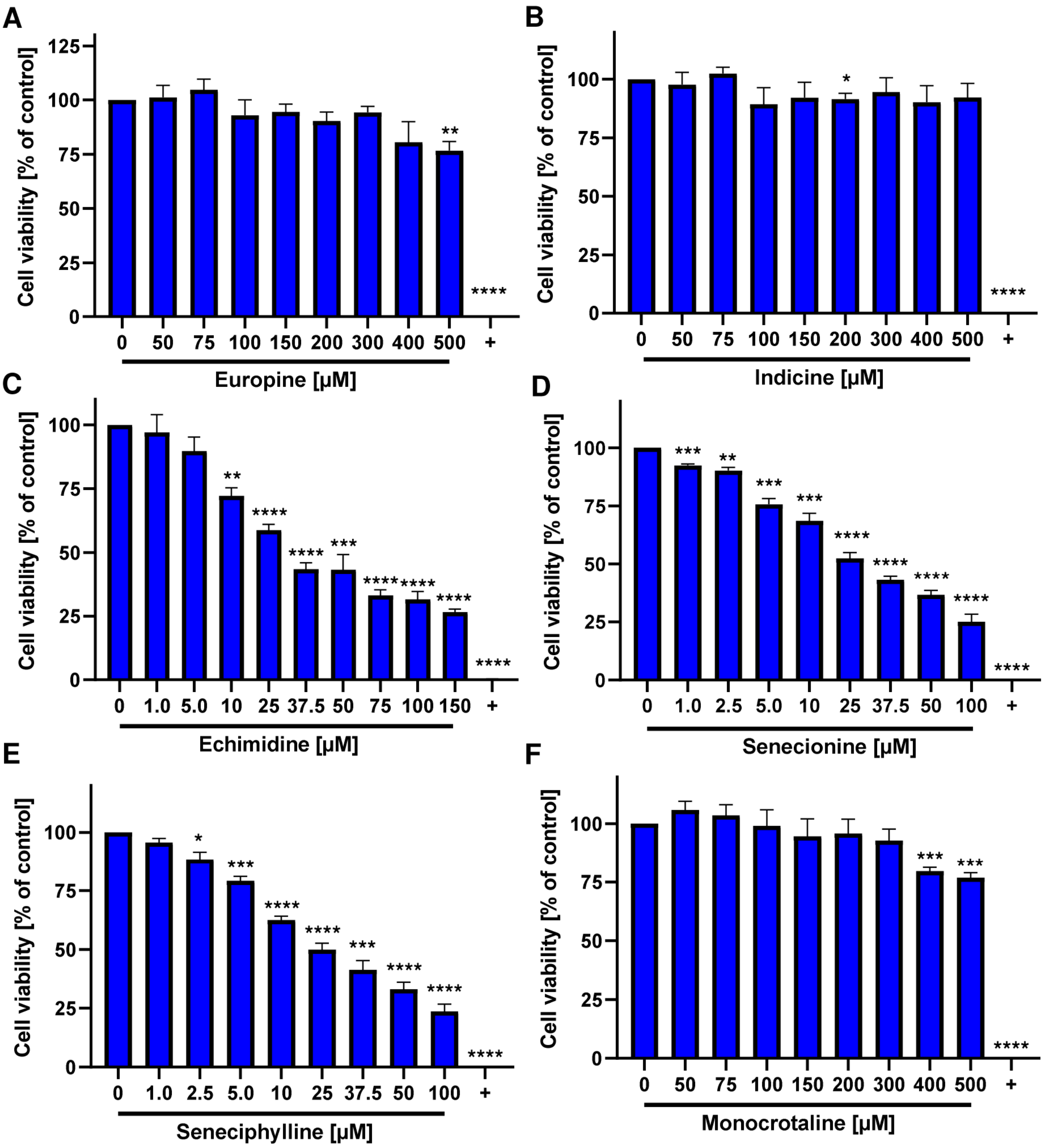

Figure S2: Structure-dependent cytotoxic effects in HepG2-CYP3A4 cells triggered by different PAs. Viability of HepG2-CYP3A4 cells 24 h after treatment with increasing concentrations of europine (**A**), indicine (**B**), echimidine (**C**), senecionine (**D**), seneciphylline (**E**), and monocrotaline (**F**)**.** Saponin was used as a positive control (+) and solvent as a negative control (0). Mean + SEM are shown for each incubation (n=3, each measured as triplicates). Statistical analyses were performed using unpaired Students t-test with respect to the negative control. "*" P≤0.05, "**" P ≤0.01, "***" P ≤0.001, "****" P ≤0.0001.

Figure S3

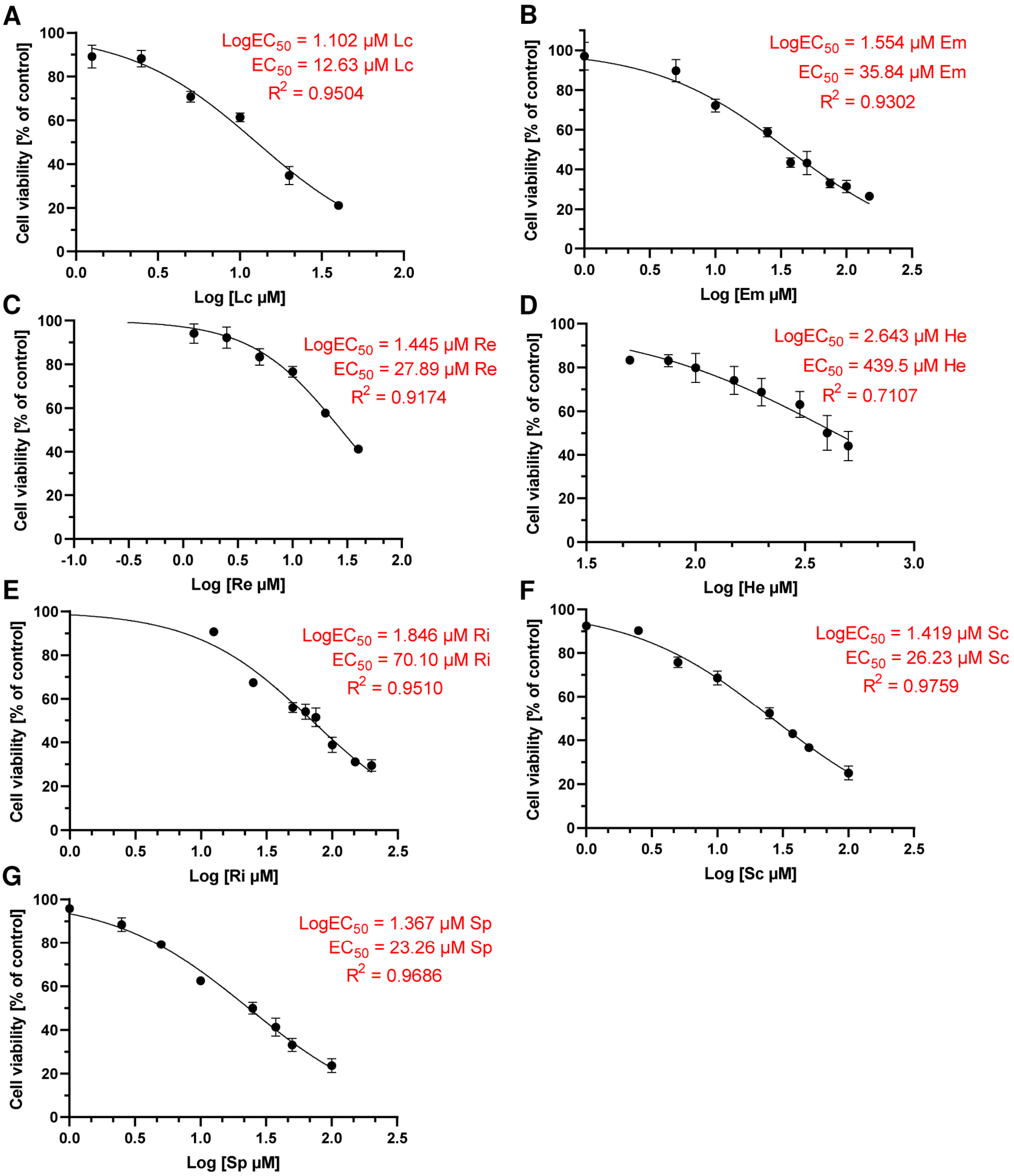

Figure S3: Determination of EC_{50} values from PA concentration-response data in HepG2-CYP3A4 cells after 24 h exposure. The data were transformed via GraphPad and fitted with a sigmoidal, non-linear model to determine the relative cytotoxicity for lasiocarpine (**A**), echimidine (**B**), retrorsine (**C**), heliotrine (**D**), riddelliine (**E**), senecionine (**F**) and seneciphylline (**G**). The relative cytotoxicity was calculated as the effective concentration, at which cell viability was reduced by 50 % (EC_{50}).

Figure S4

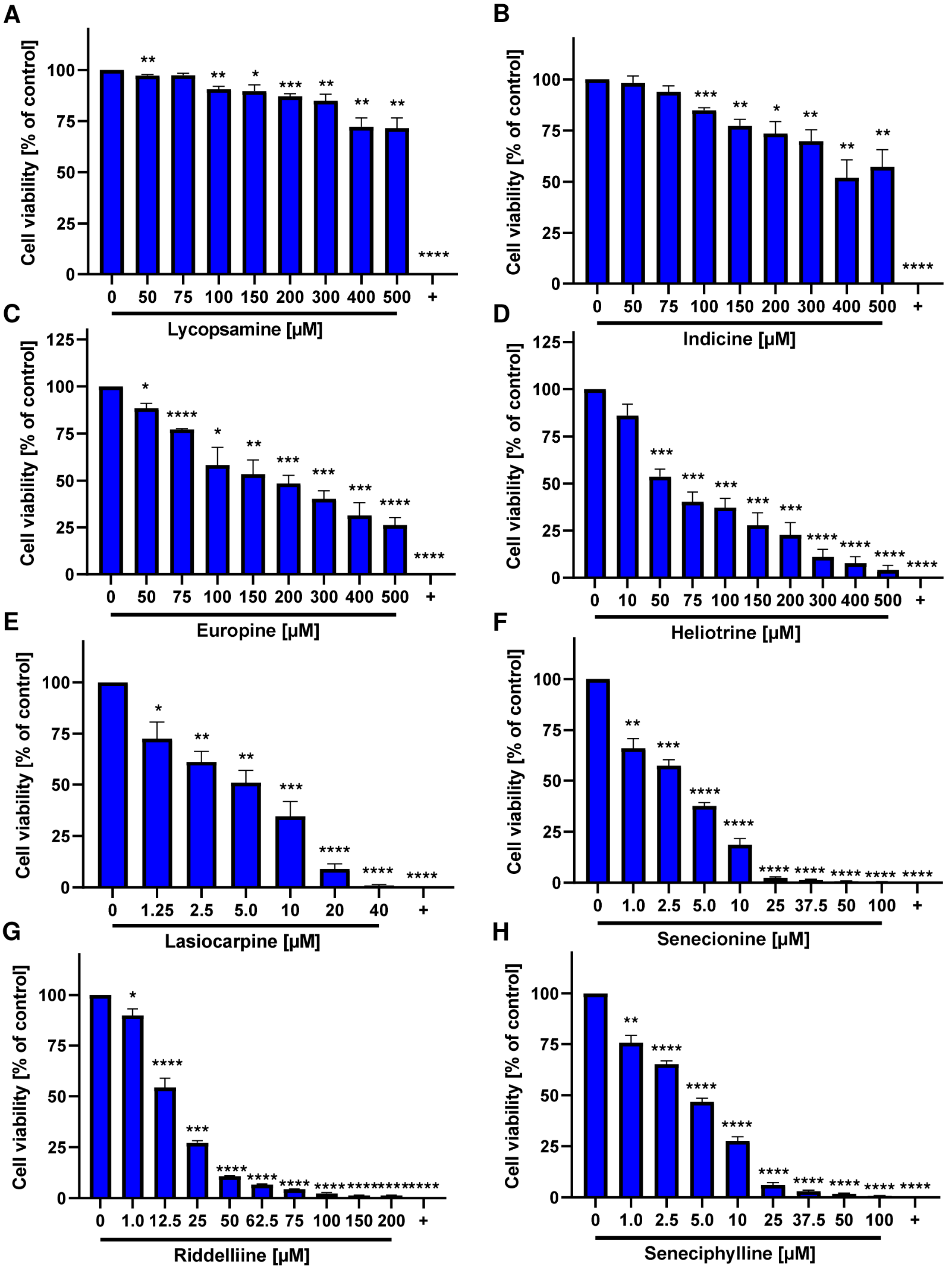

Figure S4: Structure-dependent cytotoxic effects in HepG2-CYP3A4 cells triggered by different PAs. Viability of HepG2-CYP3A4 cells 72 h after treatment with increasing concentrations of lycopsamine (**A**), indicine (**B**), europine (**C**), heliotrine (**D**), lasiocarpine (**E**), senecionine (**F**), riddelliine (**G**) and seneciphylline (**H**). Saponin was used as a positive control (+) and solvent as a negative control (0). Mean + SEM are shown for each incubation (n=3,

each measured as triplicates). Statistical analyses were performed using unpaired Students t-test with respect to the negative control. * P≤0.05, ** P ≤0.01, *** P ≤0.001, **** P ≤0.0001.

Figure S5

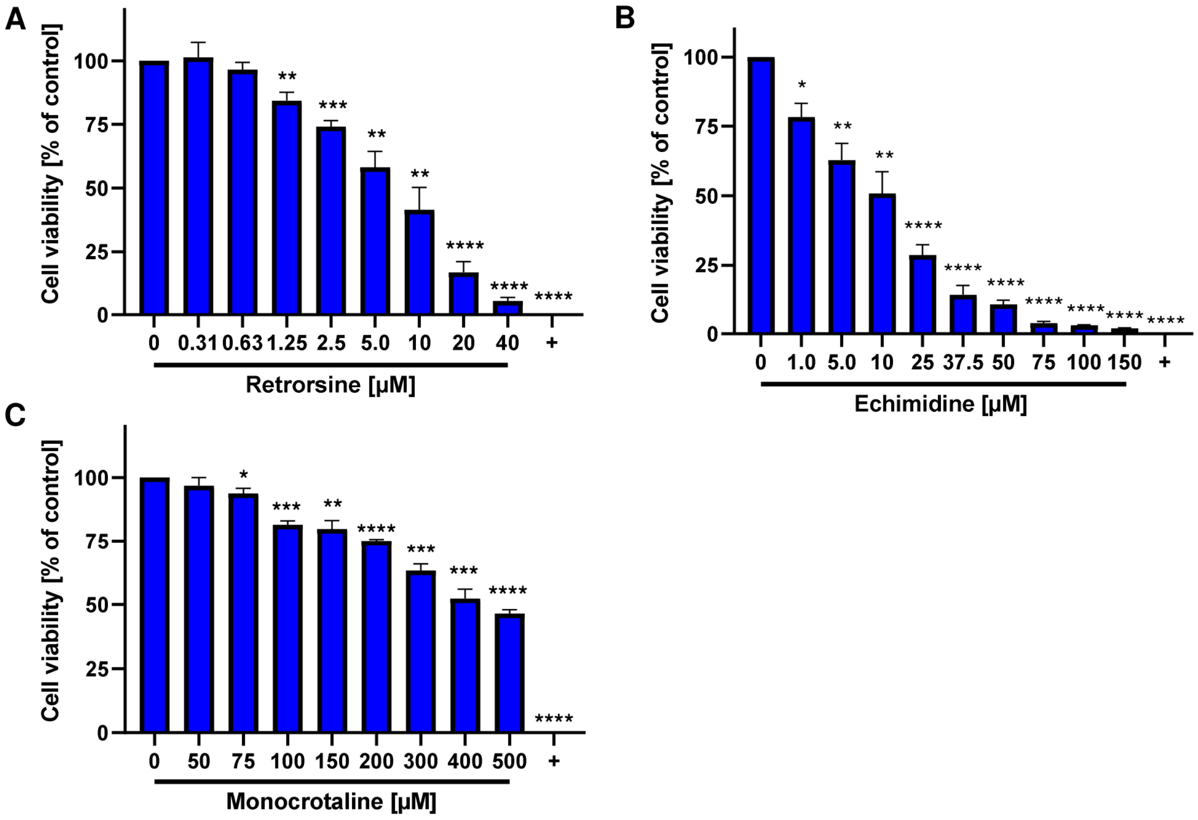

Figure S5: Structure-dependent cytotoxic effects in HepG2-CYP3A4 cells triggered by different esterified PAs. Viability of HepG2-CYP3A4 cells 72 h after treatment with increasing concentrations of retrorsine (**A**), echimidine (**B**) and monocrotaline (**C**). Saponin was used as a positive control (+) and solvent as a negative control (0). Mean + SEM are shown for each incubation (n=3, each measured as triplicates. Statistical analyses were performed using unpaired Students t-test with respect to the negative control. * P≤0.05, ** P ≤0.01, *** P ≤0.001, **** P ≤0.0001.

Figure S6

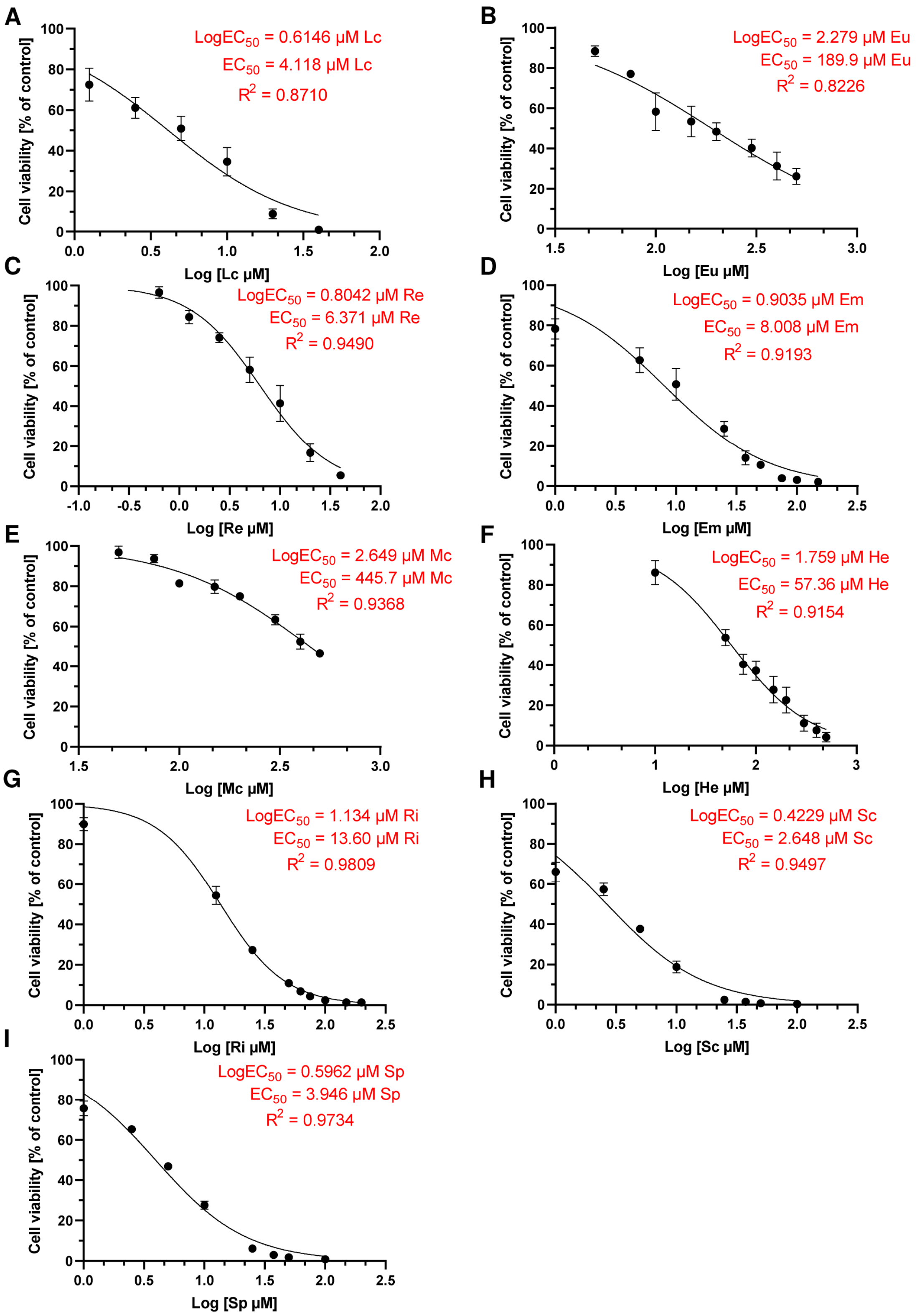

Figure S6: Determination of EC_{50} values from PA concentration-response data in HepG2-CYP3A4 cells after 72 h exposure. The data were transformed via GraphPad and fitted with a sigmoidal, non-linear model to determine the relative cytotoxicity for lasiocarpine (**A**), europine (**B**), retrorsine (**C**), echimidine (**D**), monocrotaline (**E**), heliotrine (**F**), riddelliine

(**G**), senecionine (**H**) and seneciphylline (**I**). The relative cytotoxicity was calculated as the effective concentration, at which cell viability was reduced by 50 % (EC_{50}).

Figure S7

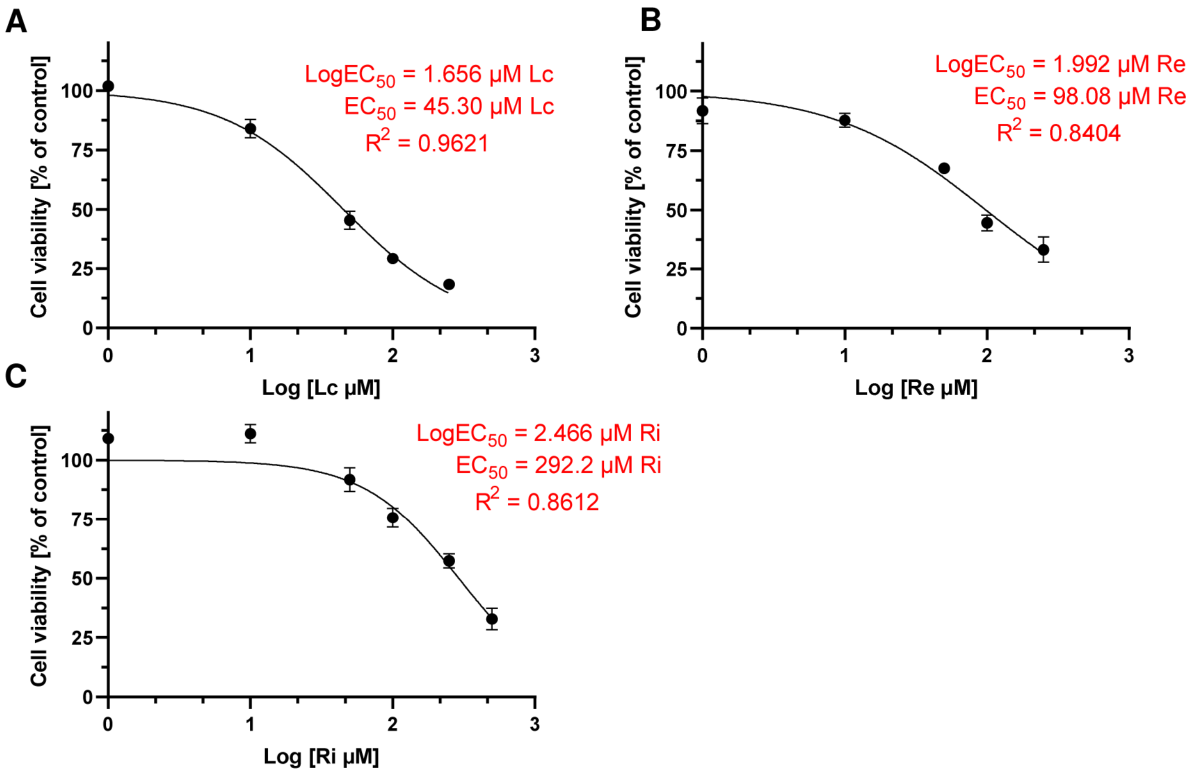

Figure S7: Determination of EC_{50} values from PA concentration-response data in primary human hepatocytes after 24 h exposure. The data were transformed via GraphPad and fitted with a sigmoidal, non-linear model to calculate the relative cytotoxicity for lasiocarpine (**A**), retrorsine (**B**) and riddelliine (**C**). The relative cytotoxicity was calculated as the effective concentration, at which cell viability was reduced by 50 % (EC_{50}).

Figure S8

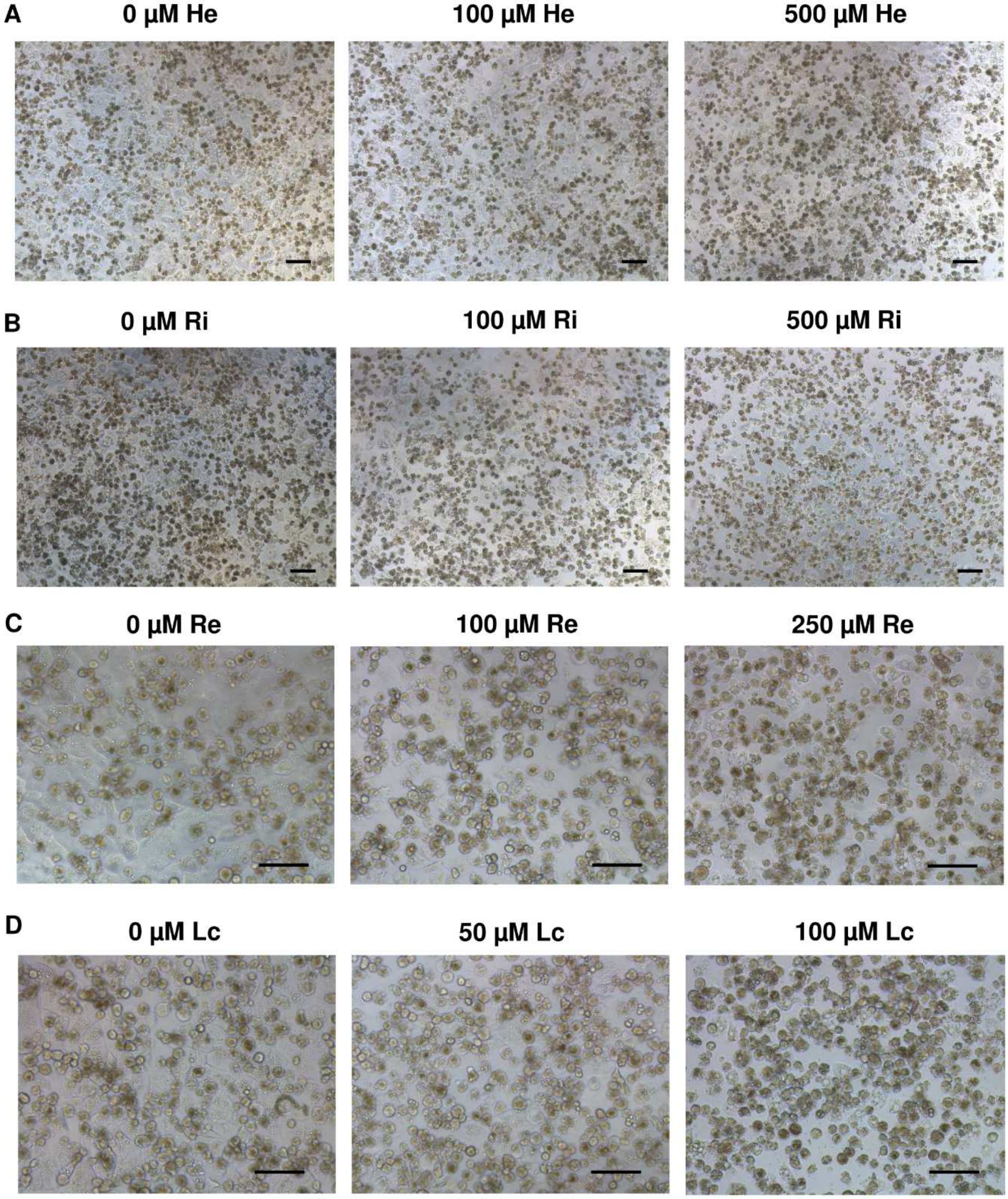

Figure S8: Cell morphology of primary human hepatocytes exposed to increasing PA concentrations. A-D: Representative microscopic images of primary human hepatocytes after 24 h incubation with different concentrations of heliotrine (**A**), riddelliine (**B**), retrorsine (**C**) and lasiocarpine (**D**). Representative images are shown. Scale bar: 100 µm.

Figure S9

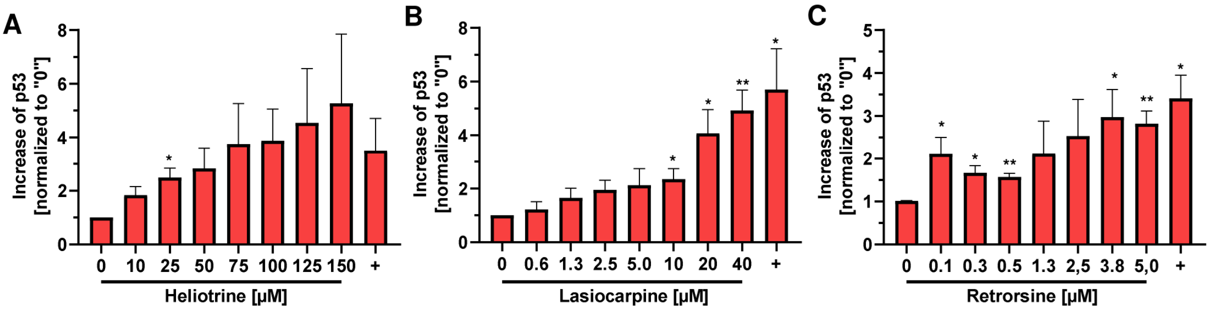

Figure S9: Structure-dependent p53 accumulation in HepG2-CYP3A4 cells triggered by different PAs. Densitometric evaluations of p53 after 24 h incubation with heliotrine (**A**), lasiocarpine (**B**) and retrorsine (**C**) in HepG2-CYP3A4. Etoposide was used as a positive (+) and solvent as a negative control (0). HSP90 served as loading control. p53 level relative to the loading control and normalized versus the negative control. Data depicted as mean + SEM (N=3). Statistical analysis was performed by unpaired Student's t test on negative control. * P≤0.05, ** P ≤0.01, *** P ≤0.001, **** P ≤0.0001.

Figure S10

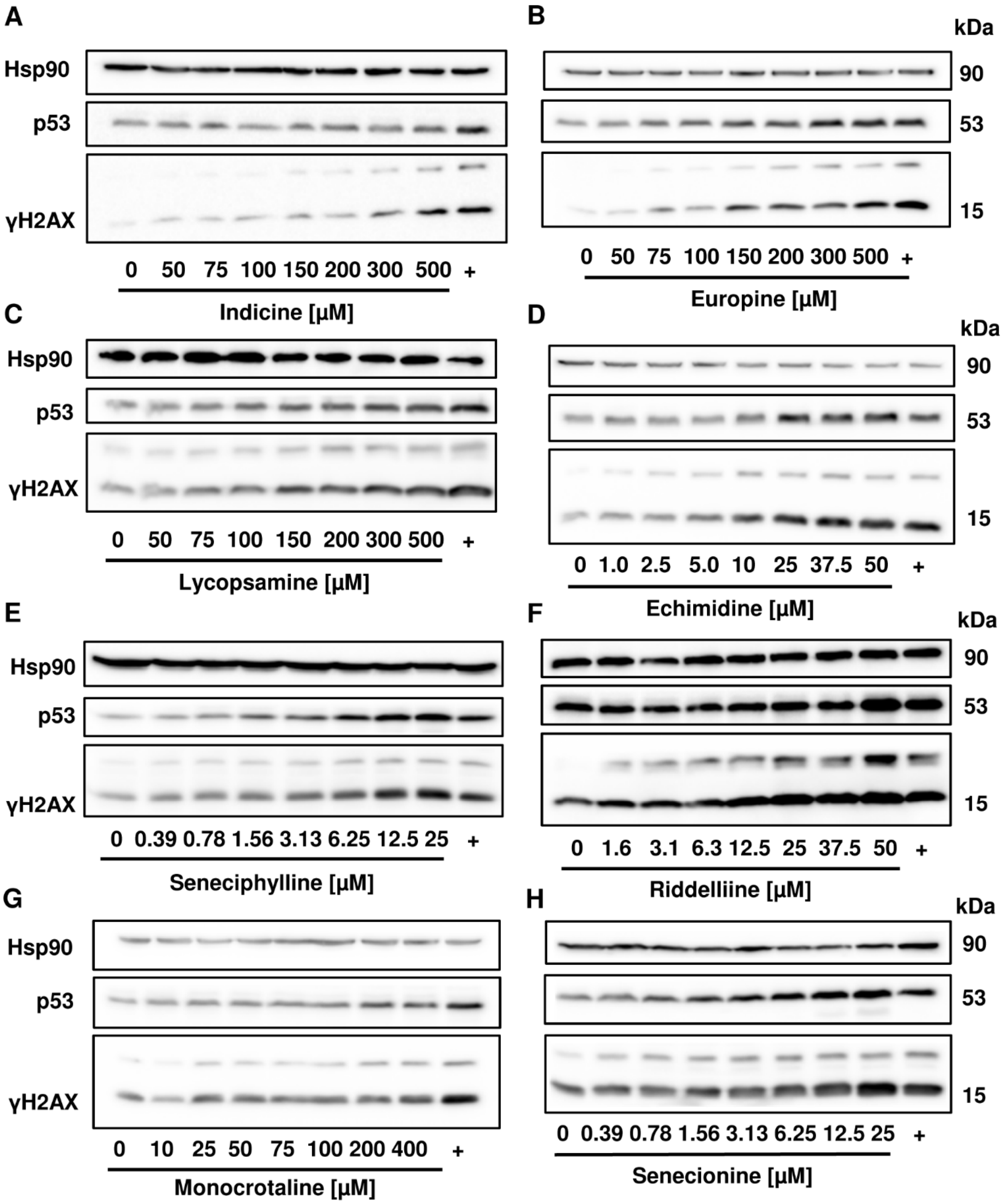

Figure S10: Structure-dependent γH2AX and p53 formation in HepG2-CYP3A4 cells triggered by different PAs. Representative western blots of γH2AX and p53 after 24 h incubation with indicine (**A**), europine (**B**), lycopsamine (**C**), echimidine (**D**), seneciphylline (**E**), riddelliine (**F**), monocrotaline (**G**) and senecionine (**H**) in HepG2-CYP3A4 cells. Etoposide was used as a positive (+) and solvent as a negative control (0). HSP90 served as loading control.

Figure S11

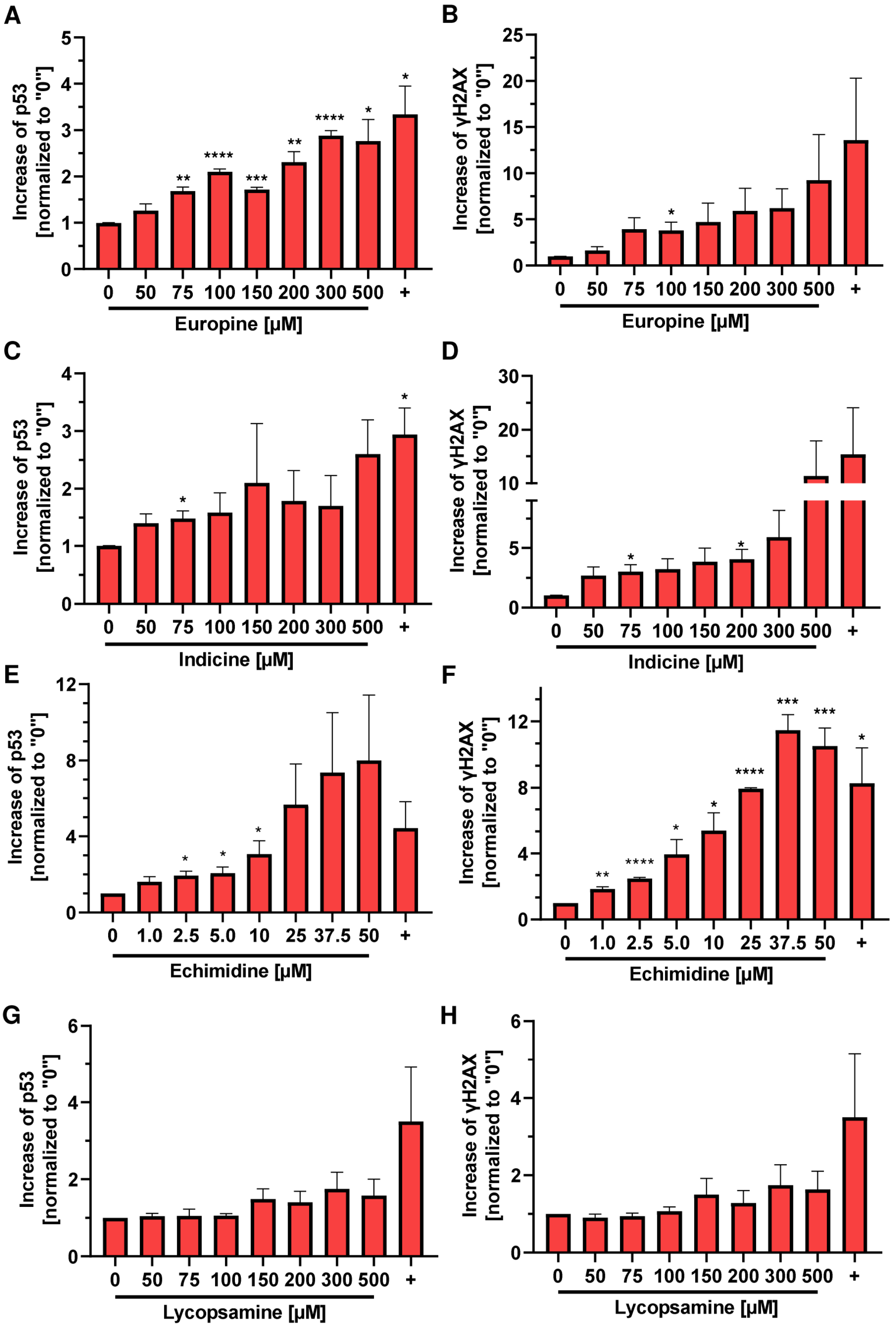

Figure S11: Structure-dependent γH2AX and p53 formation in HepG2-CYP3A4 cells triggered by different PAs. Densitometric evaluations of γH2AX and p53 after 24 h incubation with europine (**A-B**), indicine (**C-D**), echimidine (**E-F**) and lycopsamine (**G-H**) in HepG2-CYP3A4. Etoposide was used as a positive (+) and solvent as a negative control (0). HSP90

served as loading control. γH2AX and p53 levels relative to the loading control and normalized versus the negative control. Data depicted as mean + SEM (N=3). Statistical analysis was performed by unpaired Student's t test on negative control. "*" $P \leq 0.05$, "**" $P \leq 0.01$, "***" $P \leq 0.001$, "****" $P \leq 0.0001$

Figure S12

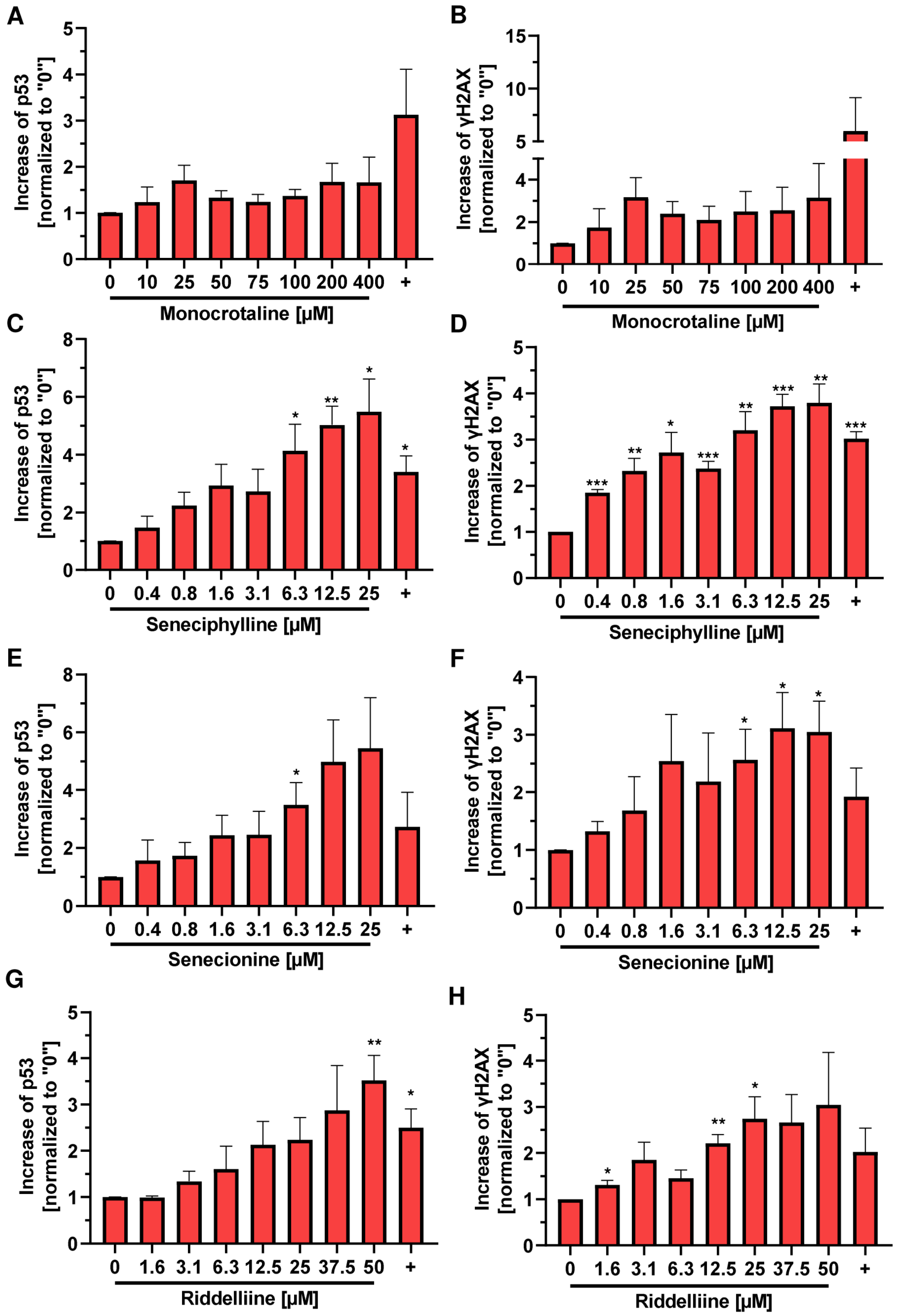

Figure S12: Structure-dependent γH2AX and p53 formation in HepG2-CYP3A4 cells triggered by different esterified PAs. Densitometric evaluations of γH2AX and p53 after 24 h incubation with monocrotaline (**A-B**), seneciphylline (**C-D**), senecionine (**E-F**) and riddelliine (**G-H**) in HepG2-CYP3A4. Etoposide was used as a positive (+) and solvent as a negative

control (0). HSP90 served as loading control. γH2AX and p53 levels relative to the loading control and normalized versus the negative control. Data depicted as mean + SEM (N=3). Statistical analysis was performed by unpaired Student's t test on negative control. * P≤0.05, ** P ≤0.01, *** P ≤0.001, **** P ≤0.0001.

Figure S13

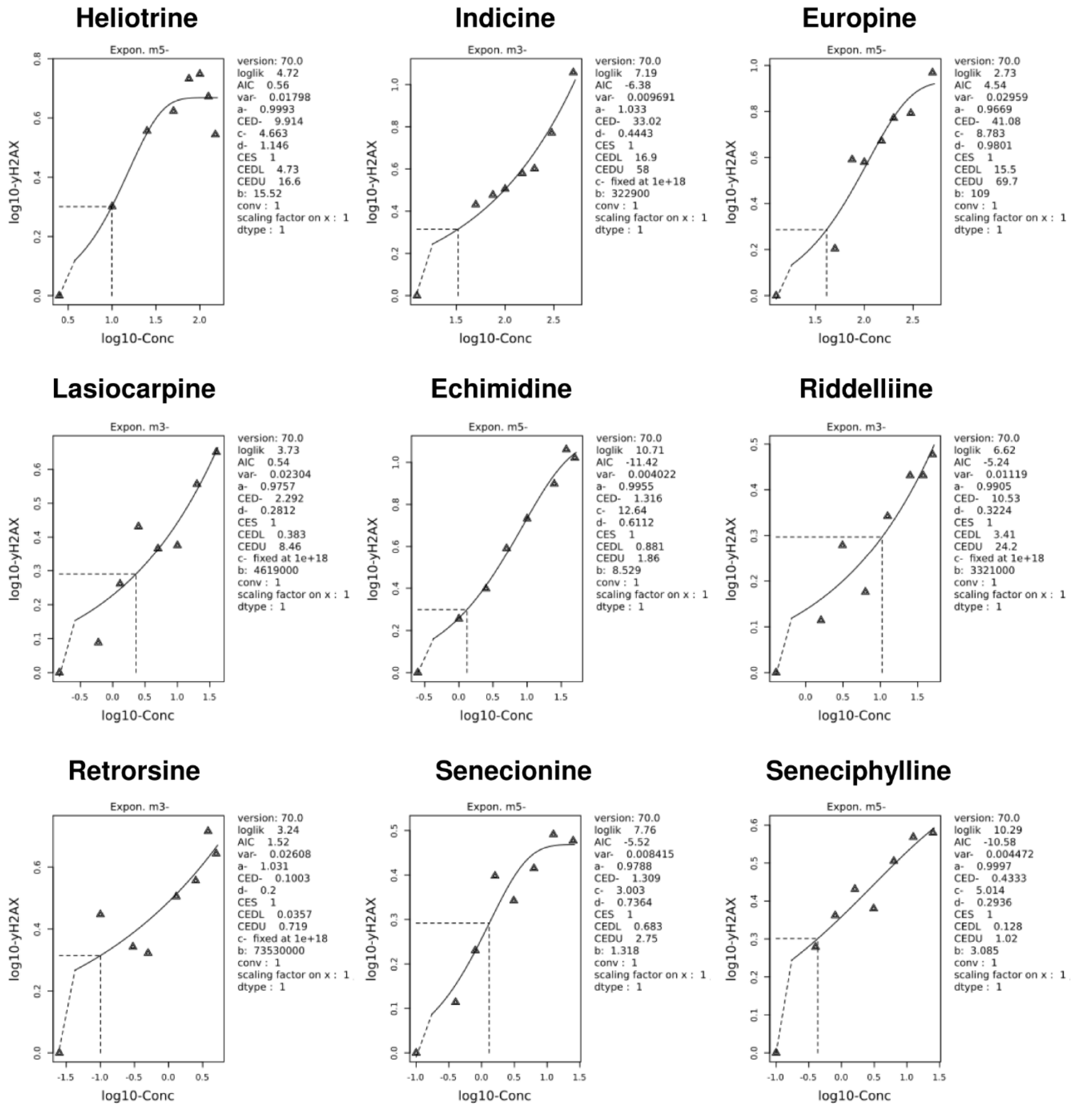

Figure S13: BMD modeling of γH2AX data from heliotrine, indicine, europine, lasiocarpine, echimidine, riddelliine, retrorsine, senecionine and seneciphylline in HepG2-CYP3A4.

Figure S14

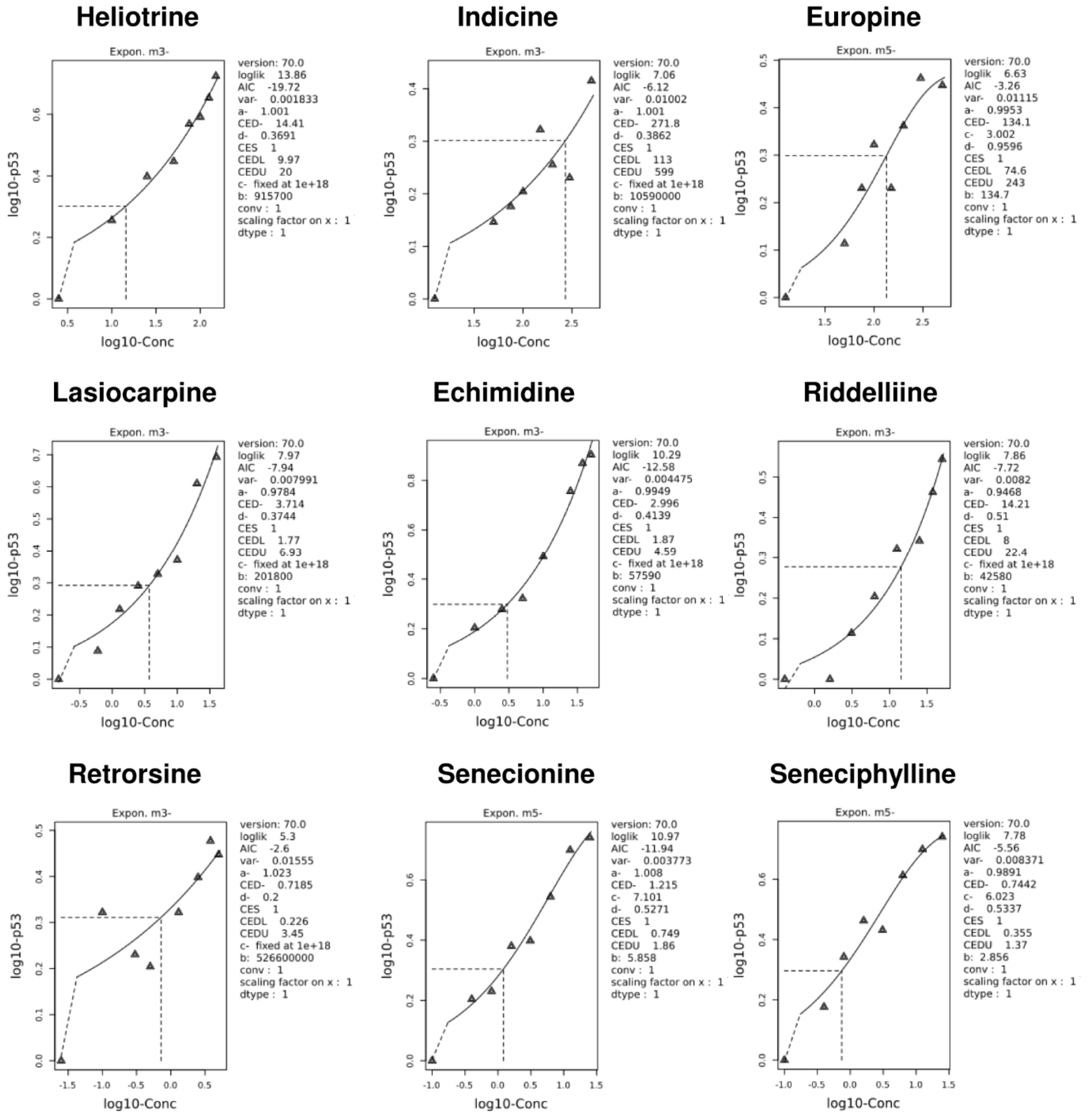

Figure S14: BMD modeling of p53 data from heliotrine, indicine, europine, lasiocarpine, echimidine, riddelliine, retrorsine, senecionine and seneciphylline in HepG2-CYP3A4.

Figure S15

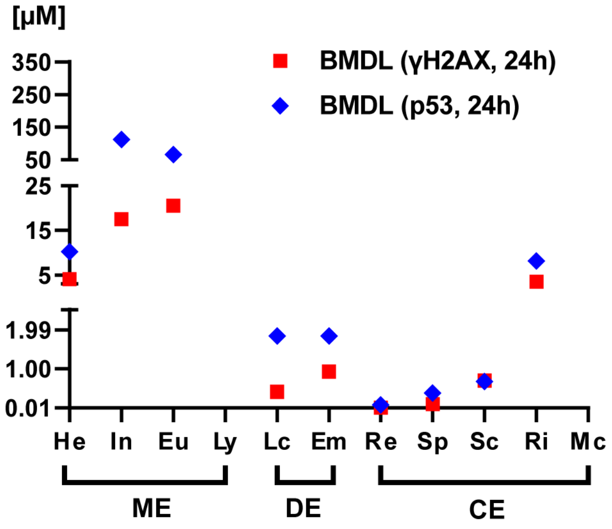

Figure S15: BMDL values for different PAs based on γH2AX and p53 data. BMD modeling was performed with concentration-response data using PROAST from EFSA with a CES of 1.0. ME: monoester; DE: open diester; CE: cyclic diester.

Figure S16

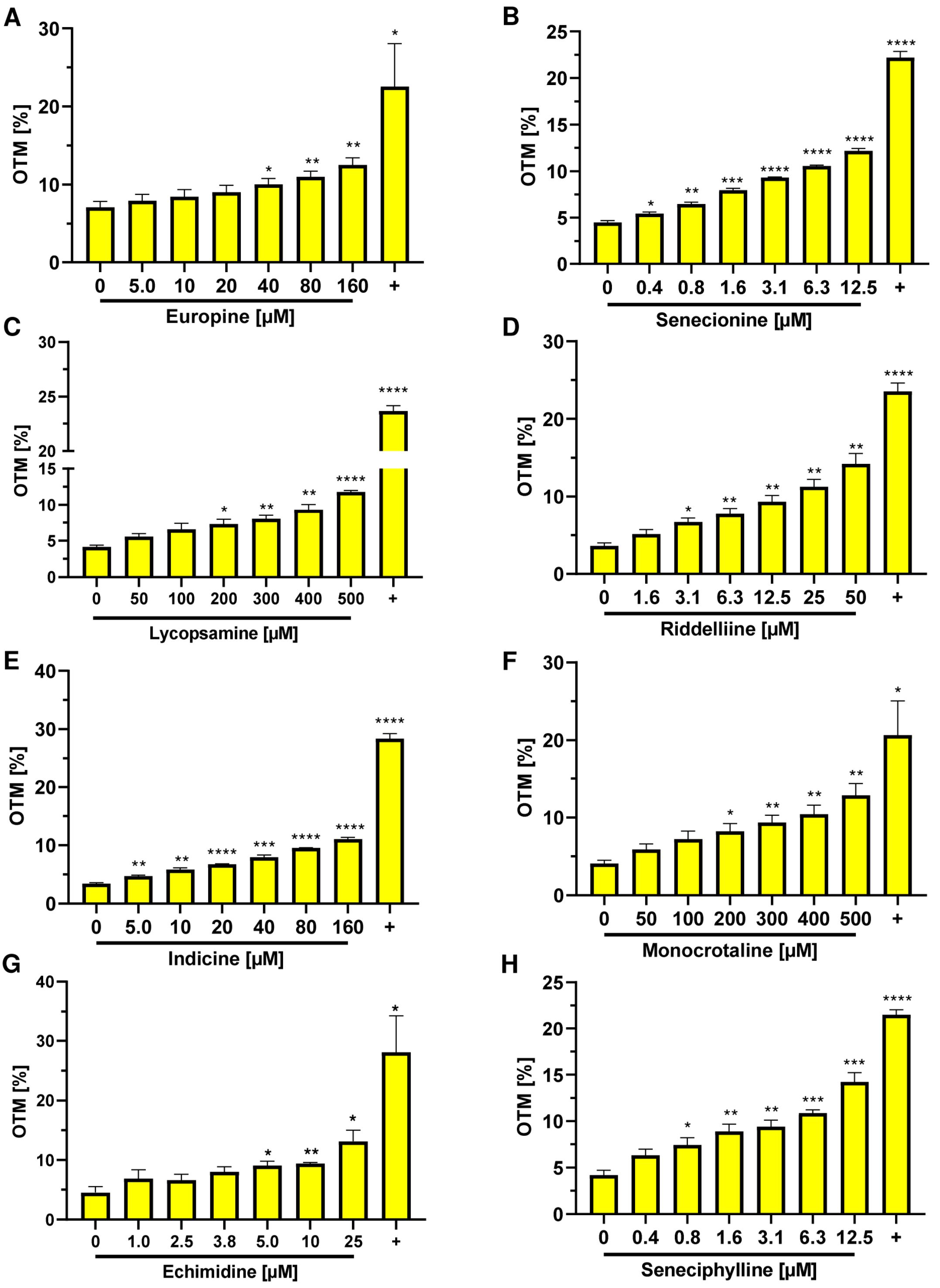

S16: Structure-dependent DNA strand break induction in HepG2-CYP3A4 by different PAs. Olive tail moments (OTM), which represents DNA strand break formation, in HepG2-CYP3A4 after 24 h incubations with europine (A), senecionine (B), lycopsamine (C), riddelliine (D), indicine (E), monocrotaline (F), echimidine (G) and seneciphylline (H). tBOOH was used as a positive (+) and solvent as a negative control (0). Data are shown as mean + SEM (N=3,

except for heliotrine and europine N=4). At least 50 comets per slide were counted. Statistical analyses were performed using unpaired two-tailed Students t-test with respect to the negative control. * $P \leq 0.05$, ** $P \leq 0.01$, *** $P \leq 0.001$, **** $P \leq 0.0001$.

Figure S17

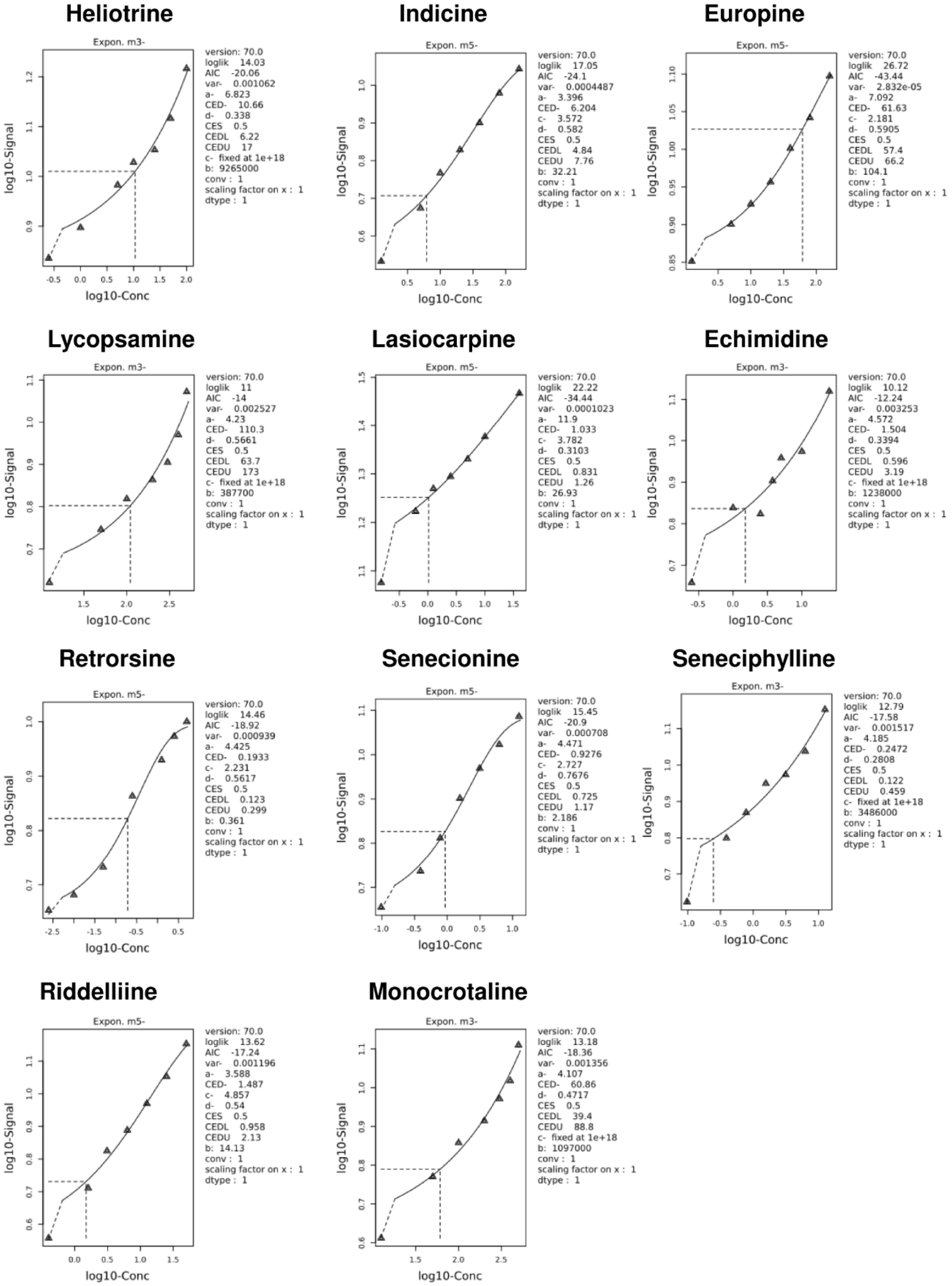

Figure S17: BMD modeling of Comet data for heliotrine, indicine, europine, lycopsamine, lasiocarpine, echimidine, riddelliine, retrorsine, monocrotaline, senecionine and seneciphylline in HepG2-CYP3A4.

Figure S18

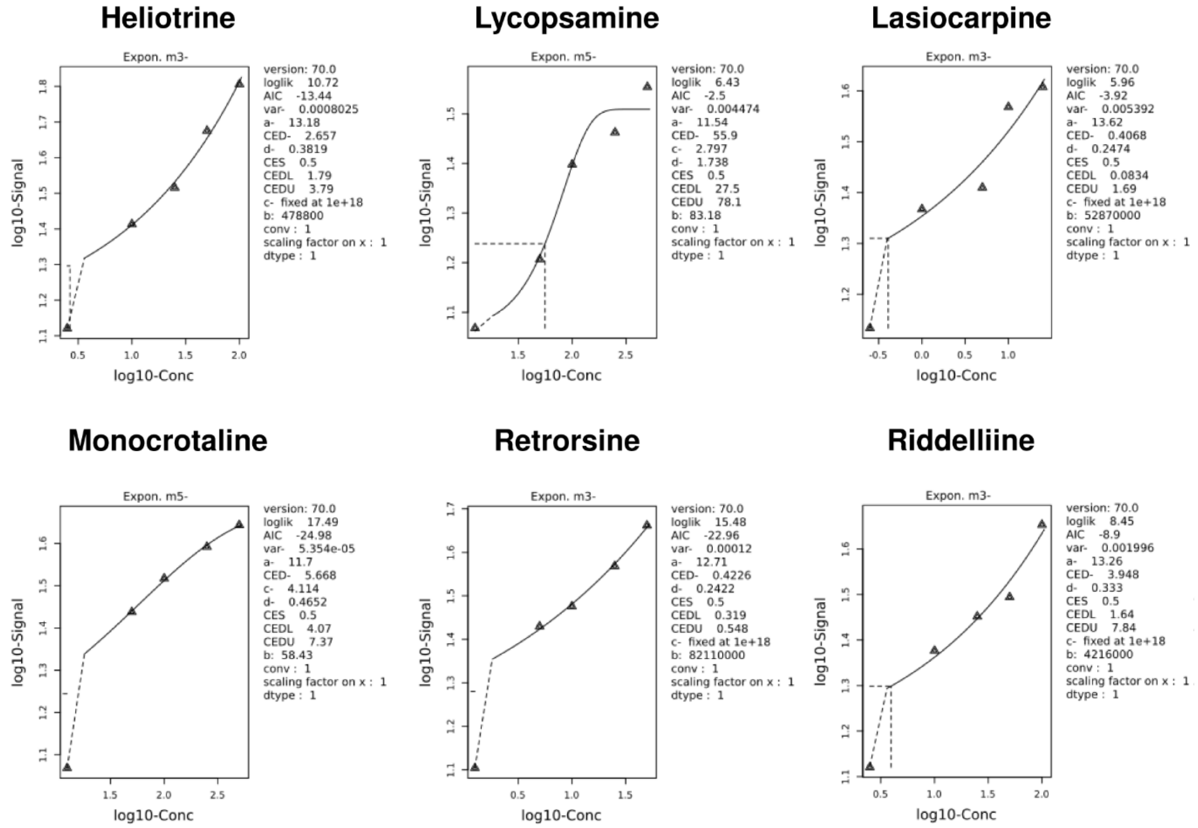

Figure S18: BMD modeling of γH2AX data for heliotrine, lycopsamine, lasiocarpine and monocrotaline, retrorsine and riddelliine in primary human hepatocytes.

Supplementary Tables

Table S1: BMD values obtained from concentration-response data for PAs in HepG2-CYP3A4. Data from γH2AX analysis for eleven PAs in HepG2-CYP3A4 cells were used for BMD modeling as described in Material & Methods. NA: not analyzable due to weak, concentration-independent effects.

PA	BMDL (γH2AX) [µM]	BMD (γH2AX) [µM]	BMDU (γH2AX) [µM]
He	4.09	8.84	16.00
Ly	NA	NA	NA
In	17.5	34.46	60.93
Eu	20.5	43.61	67.68
Lc	0.42	2.30	8.22
Em	0.93	1.36	1.89
Sp	0.10	0.36	1.01
Sc	0.70	1.27	2.57
Re	0.01	0.09	0.71
Ri	3.48	10.43	23.98
Mc	NA	NA	NA

Table S2: BMD values obtained from concentration-response data for PAs in HepG2-CYP3A4. Data from p53 analysis for eleven PAs in HepG2-CYP3A4 cells were used for BMD modeling as described in Material & Methods. NA: not analyzable due to weak, concentration-independent effects.

PA	BMDL (p53) [µM]	BMD (p53) [µM]	BMDU (p53) [µM]
He	10.2	14.64	20.30
Ly	NA	NA	NA
In	112.3	270.08	608.75
Eu	66.7	138.55	268.00
Lc	1.84	3.74	6.84
Em	1.84	3.14	4.72
Sp	0.39	0.75	1.31
Sc	0.68	1.15	1.84
Re	0.09	0.68	3.52
Ri	8.14	14.04	21.75
Mc	NA	NA	NA

Table S3: BMD values obtained from concentration-response data for PAs in HepG2-CYP3A4. Data from Comet (OTM) analysis for eleven PAs in HepG2-CYP3A4 cells were used for BMD modeling as described in Material & Methods. NA: not analyzable due to weak, concentration-independent effects.

PA	BMDL (OTM) [µM]	BMD (OTM) [µM]	BMDU (OTM) [µM]
He	6.20	10.57	16.85
Ly	62.1	109.13	173.00
In	5.26	6.39	7.63
Eu	58.28	62.10	66.10
Lc	0.84	1.03	1.25
Em	0.62	1.54	3.23
Sp	0.14	0.26	0.47
Sc	0.78	0.92	1.08
Re	0.14	0.19	0.27
Ri	1.09	1.59	2.15
Mc	39.85	61.68	90.15

Table S4: BMD values obtained from concentration-response data for PAs in primary human hepatocytes (PHH). γH2AX data from six PAs in PHH were used for BMD modeling as described in Material & Methods.

PA	BMDL (γH2AX) [µM]	BMD (yH2AX) [µM]	BMDU (yH2AX) [µM]
He	2.1	3.00	4.16
Ly	31.9	52.85	74.63
Lc	0.06	0.44	1.73
Re	0.36	0.49	0.65
Ri	1.72	4.17	8.24
Mc	6.3	8.07	9.63
